# Terahertz Modulation of Biomolecular Dynamics: Physical Mechanisms, Biological Implications, and Future Challenges

**DOI:** 10.34133/research.1432

**Published:** 2026-10-21

**Authors:** Xingrong Li, Zhihao Chen, Kang Ru, Wenhui Yang, Xuehao Wang, Lintao Huang, Baoshi Ping, Menglu Pi, Chunyu Zhu, Tingting Ren, Jingyao Dai, Ye Tian, Airong Qian

**Affiliations:** ^1^ Terahertz Biomedical Interdisciplinary Joint Research Center, Xi’an Key Laboratory of Special Medicine and Health Engineering, Key Lab for Space Biosciences and Biotechnology, School of Life Sciences and Technology, Northwestern Polytechnical University, Xi’an 710072, China.; ^2^ Air Force Characteristic Medical Center, Air Force Medical University, Beijing 100142, China.

## Abstract

With the rapid expansion of terahertz (THz) technologies across communications, imaging, sensing, and biomedicine, defining their biological effects, safety boundaries, and controlled intervention has become a critical scientific priority for their responsible deployment. Although diverse biological responses have been observed at different levels of biological organization, the findings are highly dependent on exposure parameters and biological models. Moreover, how molecular perturbations propagate across scales to produce higher-order biological effects remains poorly understood. Because biomacromolecules and their hydration environments constitute the primary interface which THz radiation interacts with biological systems, elucidating THz bioeffects at the molecular scale is essential for mechanistic understanding. Here, we systematically review recent advances in THz interactions with key biological targets, including proteins, nucleic acids, ion channels, and biological membranes, and examine how local molecular perturbations translate into higher-level phenotypes. On this basis, we critically assess the major challenges facing the field and propose an integrative framework for mechanistic validation. By consolidating dispersed findings and emphasizing methodological standardization, this review provides a coherent conceptual basis for interpreting THz bioeffects and guiding their biomedical translation.

## Introduction

Terahertz (THz) waves generally refer to electromagnetic radiation between the millimeter-wave and far-infrared regions, spanning frequencies of 0.1 to 10 THz (wavelengths of 3 to 0.03 mm) [1,2]. Situated at the interface between electronics and photonics, this spectral region combines broad bandwidth with low photon energy [3], offering distinct advantages for spectral characterization [4–6], high-speed wireless communication [7–11], and nondestructive testing [12–15]. As wireless data traffic continues to grow, the THz band has emerged as a key spectral resource for sixth-generation wireless networks (6G) [7,8,11]. Meanwhile, advances in low-loss fibers, waveguides, antenna arrays, sources, and integrated modulators are enabling more efficient THz generation, transmission, delivery, and control [7,10,16,17]. Combined with multiplexing, intelligent signal processing, and machine learning, these technologies are accelerating the integration and intelligent operation of THz systems, although challenges remain in microfabrication, material loss, device uniformity, coupling efficiency, and system-level integration.

As efficient sources, detectors, transmission media, and control devices mature, the conventional “THz gap” is gradually narrowing, accelerating the transition of THz technologies from laboratory systems to communication, portable, and biomedical platforms [2,16,18–22]. This broader deployment increases potential biological exposure and heightens safety concerns. Importantly, THz frequencies overlap with biomolecular vibrations, conformational fluctuations, and dielectric relaxation to biomolecules and their hydration environments, THz radiation may therefore alter biomolecular dynamics and interaction networks through dielectric, water-mediated, or frequency-selective coupling, with these perturbations subsequently propagating to higher-order processes such as cellular signaling, metabolic homeostasis, and tissue function [23–28].

To better capture the biophysical relevance of electromagnetic interactions, some studies have extended the frequency range under consideration to as high as 100 THz, thereby encompassing a broader spectrum of molecular vibrations and hydration dynamics [19,29,30]. Investigations have been conducted across multiple levels of biological organization, from biomolecules and subcellular structures to individual cells, tissues, and animal models, suggesting that THz radiation may influence biological processes over a wide range of spatial scales [27,31–36]. However, substantial differences in experimental platforms and irradiation doses have limited cross-study comparability, resulting in a fragmented body of evidence and hindering a comprehensive understanding of THz bioeffects [37–40]. Moreover, conflicting experimental results have sparked debate about whether THz radiation, despite its low photon energy, can adversely affect biological systems, further complicating the establishment of unified safety standards and hindering clinical translation [41,42].

Given the complexity of biological systems, macroscopic observations are often insufficient to identify primary targets or resolve underlying mechanisms. As a result, an increasing number of studies are shifting toward molecular- and cellular-level investigations to achieve a more accurate understanding and application, as illustrated in Fig. 1. In this review, we examine the field from both biological and physical perspectives, integrating theoretical modeling and experimental progress, to systematically review the research advancements in THz bioeffects at the molecular level. It also discusses the current challenges and future directions, with the aim of providing theoretical foundations and practical guidance for the in-depth study and clinical application of THz technology in the biomedical field.

**Fig. 1.**
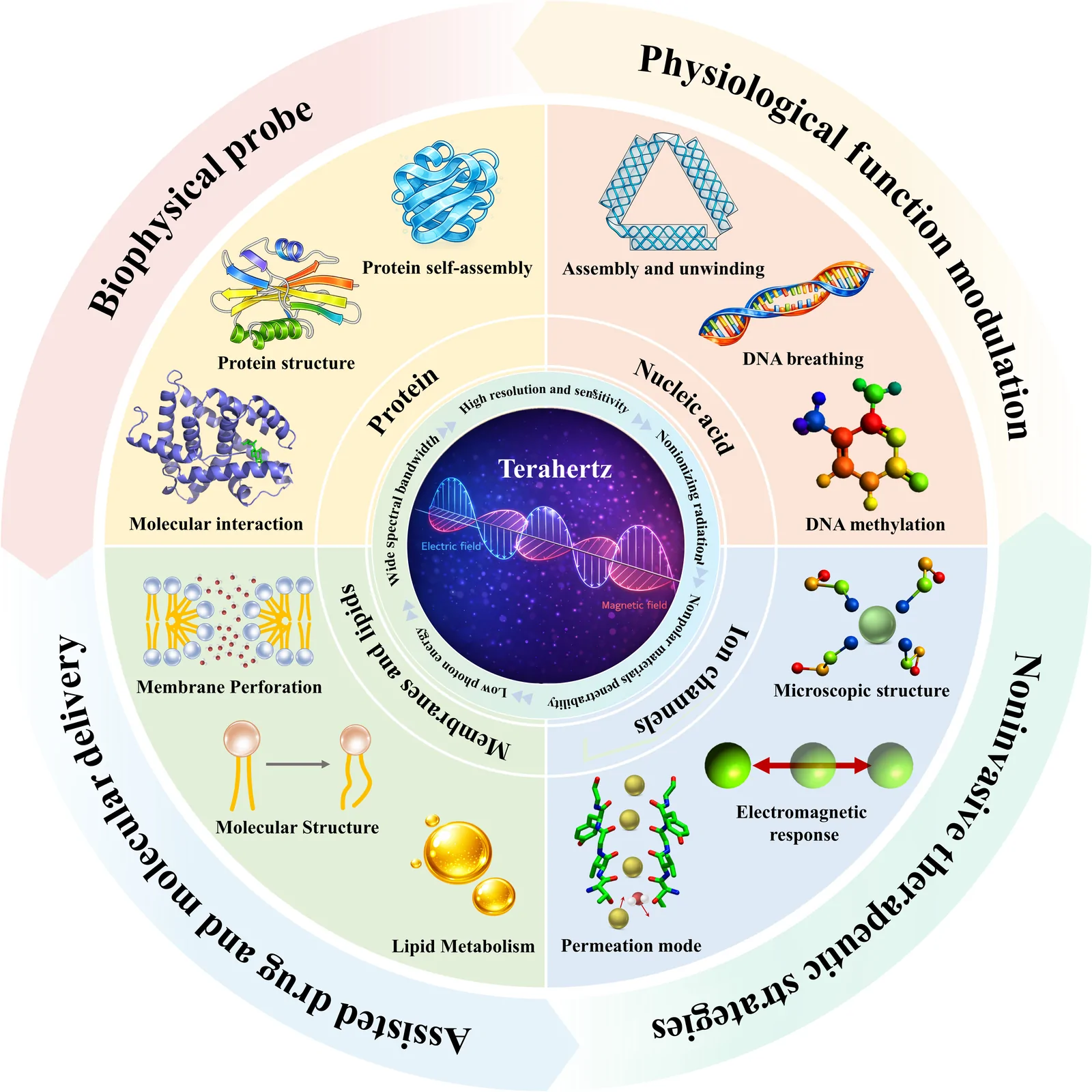
Overview of terahertz (THz) bioeffects from a molecular perspective. THz radiation combines low photon energy, nonionizing properties, broad spectral bandwidth, and high penetration (especially in nonpolar materials) with high resolution and sensitivity. It has been reported to modulate the structure and dynamics of proteins, nucleic acids, ion channels, membranes, and lipids and is being explored as a nonthermal modality for biophysical probing, physiological modulation, noninvasive therapy, and drug delivery.

## Foundations of THz Bioeffects

### Low-frequency collective motions and noncovalent interactions

A fundamental consensus is that the 3-dimensional structures of biomolecules, together with their multiscale dynamics, provide the physical basis for biological function. At the molecular scale, when an external field overlaps with intrinsic molecular vibrations, local conformational motions or collective modes may be resonantly excited, enabling efficient transfer of energy into internal molecular motions and selectively enhancing particular dynamical channels [43–46]. Unlike higher frequency radiation, which primarily excites electronic transitions or chemical bonds, THz radiation preferentially couples to low-energy excitations (Fig. 2B), such as dipolar relaxation, bond torsion, molecular vibrations, and collective motions, providing a window into dynamics from biomolecules to hydrated tissues [47–49].

**Fig. 2.**
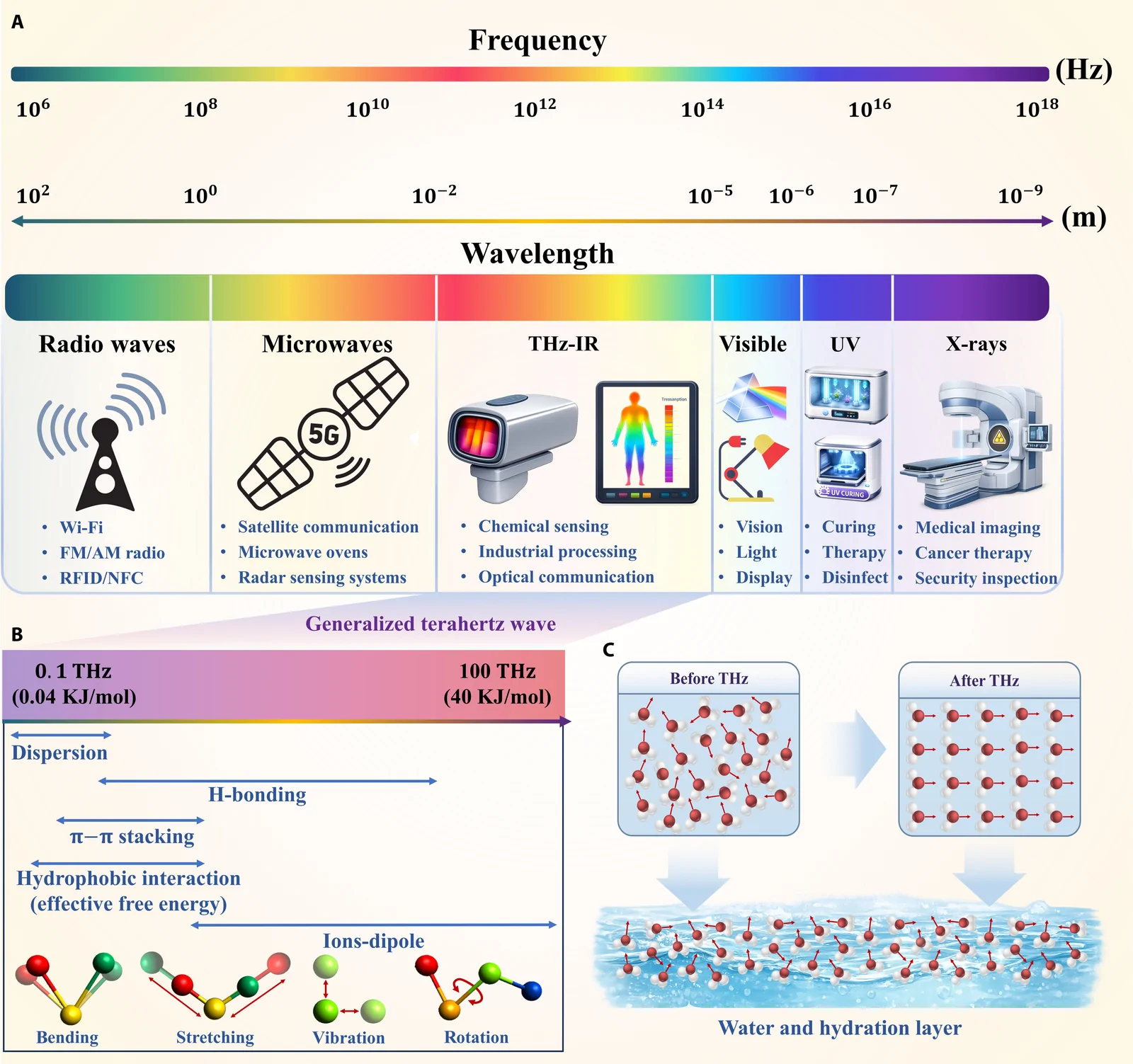
Terahertz (THz) spectrum and its coupling to molecular dynamics in biological systems. (A) The THz spectrum lies between the millimeter-wave and far-infrared regions, spanning frequencies from 0.1 to 100 THz. (B) The THz frequency range overlaps with low-frequency molecular vibrational modes in biological systems, enabling selective coupling that can modulate noncovalent interaction networks without directly disrupting covalent bonds. (C) THz radiation can induce reorganization of hydration layers and trigger nonthermal responses analogous to hydration relaxation, modulating the structure and dynamics of interfacial water. FM, frequency modulation; AM, amplitude modulation; RFID, radio frequency identification; NFC, near-field communication; IR, infrared; UV, ultraviolet.

Under a broadly defined THz framework extending to 100 THz, the accessible motions also include local intramolecular vibrations of molecular backbones, side chains, and functional groups. With increasing frequency, the dominant coupling pathway generally shifts from dipolar relaxation, hydration-shell dynamics, and macromolecular collective motions toward intermolecular vibrations and localized intramolecular modes. Correspondingly, energy transfer pathways evolve from slow dipolar relaxation and hydration-shell reorganization to rapid vibrational energy redistribution and subsequent dissipation through the surrounding hydration environment. Accordingly, as shown in Table 1, we operationally divide the THz range into 4 regimes according to the dominant coupling target and energy-transfer pathways and compare their mechanisms and interpretative limits. Importantly, many mechanistic interpretations summarized here are currently based primarily on theoretical calculations and molecular dynamics simulations, with limited quantitative experimental validation. These regimes are intended to highlight mechanistic trends rather than define rigid spectral boundaries.

**Table 1.** A segmented comparison of intramolecular interaction mechanisms in the 0.1- to 100-THz range

Frequency	Dominant coupling	Energy dissipation	Interpretative limits	Refs.
0.1–1 THz	Dipolar relaxation; protein-hydration motions; membrane polarization; ionic responses; global conformational fluctuations	Periodic torque drives molecular dipoles; energy dissipates through dipolar relaxation and hydration-shell reorganization.	Broad, overlapping absorption prevents assignment to single modes; bulk heating and hydration changes must be excluded.	[27,67,87,143,187]
1–10 THz	Hydrogen-bond fluctuations; backbone torsions; collective protein and nucleic acid modes; protein–ligand motions	Energy is redistributed through anharmonic coupling and molecular interactions, then transferred to water motions.	Collective modes are strongly damped and overlapping; high-field simulations may not represent experimental exposures.	[89,103,116,121,123,188]
10–30 THz	Water librations; backbone and side-chain bending; coupled vibrations of confined water and functional groups	Excitation propagates through hydrogen-bond networks and rapidly dissipates through rotational and translational motions.	Strong water absorption obscures local field strength and heating; macromolecular evidence remains largely computational.	[101,48]
30–100 THz	Local vibrations of nucleobases, carbonyls, carboxylates, aromatic rings, and side chains	Local vibrational energy redistributes to adjacent modes and the hydration shell, with partial conversion to heat.	Overlap with the mid-infrared region complicates attribution; effects more likely reflect complex dissociation and noncovalent-network reorganization than direct bond cleavage.	[98,99,111,112,133]

THz, terahertz

These modes are strongly damped and many-body coupled, with their dynamics shaped by coordinated noncovalent interactions in the hydration environment. Local THz-induced perturbations may propagate through anharmonic coupling or molecular interactions to neighboring groups, hydration layers, and larger-scale conformational motions, thereby biasing conformational populations and reorganizing noncovalent interaction networks [50,51]. Although individual noncovalent interactions are typically 1 to 2 orders of magnitude weaker than covalent bonds and remain highly dynamic and reversible, their cooperative organization collectively stabilize biomolecular structure and function [52,53]. Dynamic reorganization of these networks, and the resulting redistribution of the energy landscape, may therefore represent a common downstream process underlying molecular responses across the THz spectrum [54,55].

### Dual roles of water and hydration layers

Water is highly responsive to THz radiation and therefore acts both as the principal energy-absorbing medium and as a potential mediator of biological effects. In confined environments, such as ion-channel filters and receptor pockets, water exhibits heterogeneous and anisotropic dielectric relaxation that differs from bulk behavior [56,57]. It is therefore often approximated as bulk-like free water and interface-constrained bound water, although no sharp boundary exists [56,58,59]. This heterogeneity underlies the dual role of water in THz-biological interactions.

Strong absorption by free water causes local heating and limits penetration, whereas hydration-water dynamics may respond to THz fields, allowing interfacial water to act as an important coupling medium between external-field perturbations and biomolecular dynamics, as shown in Fig. 2C. Because interfacial properties and the involvement of water differ across biological systems, hydration layers can contribute through distinct mechanisms. In proteins, hydration layers primarily modulate conformational dynamics and molecular recognition. In nucleic acids, the charged phosphate backbone and its hydration environment jointly shape nucleic acid dynamics, particularly processes such as molecular assembly and local breathing. In ion channels, confined water directly participates in ion dehydration, coordination, and rehydration, thereby modulating ion permeation. Therefore, distinguishing local heating from water-mediated nonthermal reorganization across relevant spatial and temporal scales remains a central challenge and a major obstacle to clinical translation [57,59,60].

In recent years, various technical strategies have been developed to mitigate the strong absorption of THz radiation by water. Microfluidic thin layers, liquid jets, and THz attenuated total reflection techniques can effectively shorten the water propagation path and reduce bulk water background [61,62]. Metasurfaces and near-field structures can further enhance the detectability of weak biomolecular responses through localized field enhancement [63,64]. However, these approaches often rely on specific sample configurations or localized field enhancement conditions, and their applicability to complex tissues and in vivo systems requires further validation. Time-resolved techniques, including pump-probe spectroscopy, the THz Kerr effect, and 2-dimensional THz spectroscopy, can distinguish ultrafast hydration dynamics from slower thermal diffusion by exploiting differences in response time, phase, and polarization [60,65,66]. Nevertheless, these methods require stringent experimental conditions, high signal-to-noise ratios, and advanced instrumentation and currently remain primarily used for mechanistic investigations.

In bioeffects studies, a more immediate challenge is to control or disentangle thermal contributions arising from water absorption. Common approaches include temperature-matched controls, real-time temperature monitoring, and heat-transfer modeling to estimate local energy deposition [67–70]. In addition, enlarging the irradiation area, increasing the source-to-target distance, or selecting biological processes that are insensitive to small temperature variations can provide important controls. For example, a study of 0.46-THz-induced actin polymerization combined temperature calculations, infrared thermometry, and temperature-dependent controls and showed a temperature increase below 0.1 °C, thereby reducing the likelihood of a purely thermal explanation [67]. However, these control strategies cannot completely exclude localized transient thermal effects. Particularly under high-peak-power pulsed irradiation, water-mediated thermomechanical effects may still contribute to biological responses [69]. Therefore, low bulk temperature elevation alone should not be considered sufficient evidence for a nonthermal mechanism.

Reducing tissue water content represents another potential strategy to improve THz transmission. Freezing can decrease the proportion of liquid water but also alters hydration structures and biomolecular dynamics through ice formation, limiting its relevance to physiological conditions [58,71]. Hyperosmotic tissue-clearing agents, such as glycerol, can enhance THz transmission in ex vivo tissues [72]; however, dehydration and osmotic stress may themselves perturb cellular states, necessitating appropriate agent-only controls. Another potentially more translatable approach is to shorten the aqueous propagation distance between the radiation source and target, for example, by using low-absorption substrates, skull thinning, or fiber-based approaches and implanted cannulas for near-target delivery [31,73]. Nevertheless, these strategies require systematic evaluation of invasiveness, safety margins, and local tissue dosimetry. Overall, although existing approaches can improve the interpretability and mechanistic attribution of THz bioeffects, they often involve alterations in the native hydration environment, propagation pathways, or local energy distribution, thereby introducing additional experimental variables. Achieving a balance between reducing water-related interference and preserving biological authenticity remains a major challenge for understanding THz bioeffects.

Theoretical modeling advances are also improving mechanistic discrimination. Classical molecular dynamics can estimate THz absorption from dipole autocorrelation functions, but fixed-charge force fields poorly capture polarization, proton transfer, and coupled solute-water electronic responses [74,75]. Emerging approaches combine first-principles molecular dynamics, nuclear quantum effects, and machine learning-accelerated simulations with modal, spatial, and cross-correlation decompositions to resolve the respective contributions of solutes, water, and their coupling [76–78]. These developments are moving computational studies beyond equilibrium spectral calculations toward explicit simulations of systems driven by realistic external THz fields.

### Experimental platforms for THz bioeffects research

Due to the broad spectrum and low photon energy of THz radiation, its effects on biological systems often involve both picosecond-scale dipole/dynamic responses and longer time-scale processes such as energy deposition, thermal diffusion, and biological adaptation [67,79,80]. These effects may present complex, multilevel coupled phenomena. Therefore, establishing a verifiable causal link between physical perturbations and biological phenomena requires platforms with controllable parameters, stable environmental conditions, and traceable dosimetry [81]. It is important to emphasize that whether THz effects should be considered to “nonthermal mechanisms” fundamentally depends on the dynamic balance between energy deposition and system heat dissipation, and careful consideration is needed to assess whether observed phenotypes can be fully explained by temperature changes [3,82,83].

As shown in Fig. 3, the THz biological effect platform should not only focus on the radiation source but also incorporate key components such as irradiation control, dosimetry monitoring, and environmental control to ensure that exposure conditions are controllable, traceable, and comparable across studies. In practical research, a single temperature rise threshold should not be used as the sole criterion for “nonthermal” effects [84]. In situ temperature monitoring and isothermal control experiments should be considered, and heat conduction modeling may be introduced when necessary to identify local hotspots and temperature measurement blind spots. Accordingly, standardized reporting of key dosimetry and platform parameters is essential, including radiation frequency (or central frequency and bandwidth), peak and average power density, irradiation area and its spatial distribution, exposure duration, as well as temperature measurement methods, measurement locations, and temporal resolution [85]. This approach will enhance research comparability, clarify the dose–effect relationships, and provide traceable evidence for safety assessment and potential applications.

**Fig. 3.**
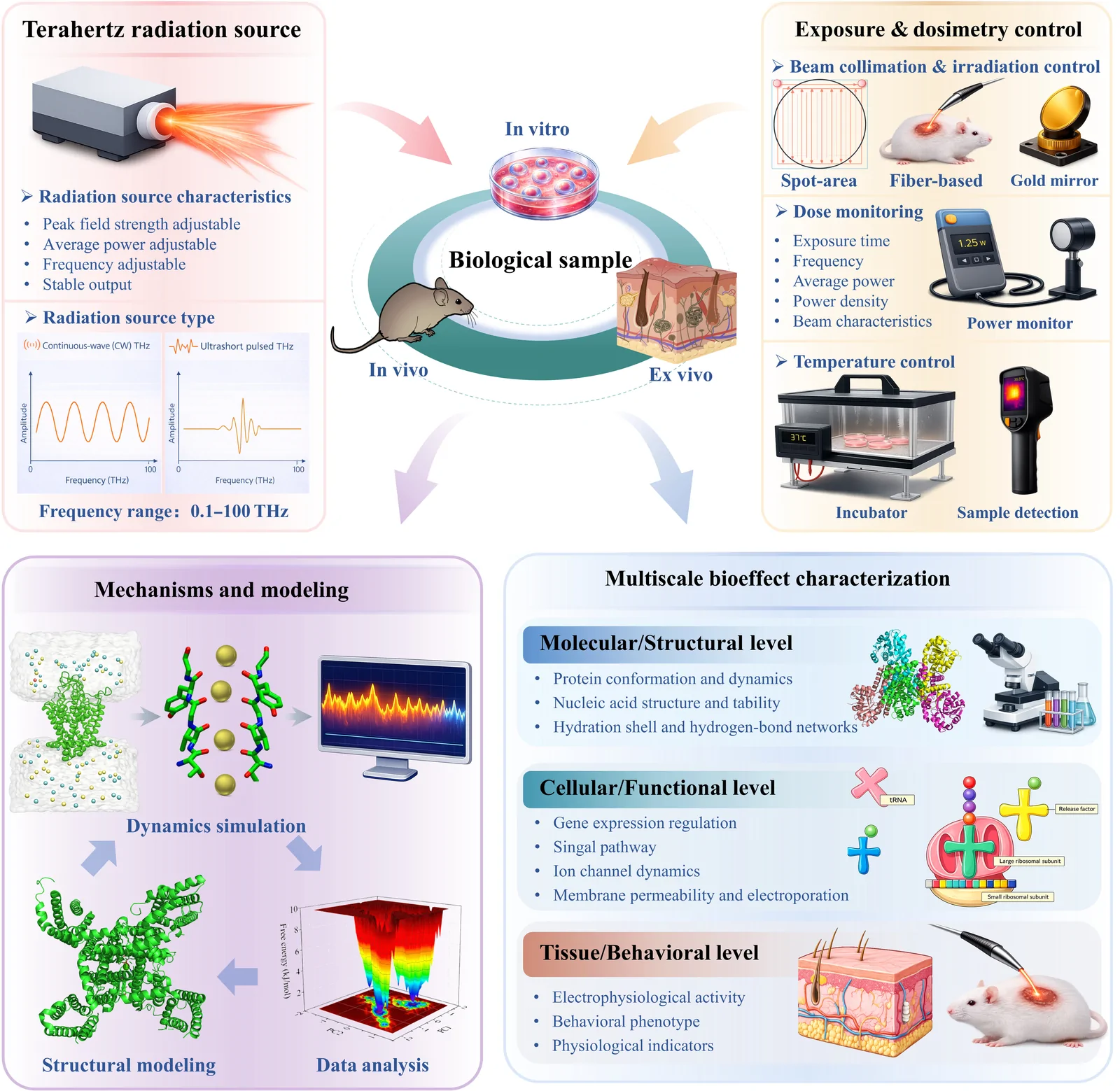
Schematic of a terahertz (THz) bioeffects research platform and multiscale investigation framework. The platform comprises a THz radiation source (continuous-wave or pulsed) and an exposure and dosimetry control system, including modules for beam collimation, irradiation control, dose monitoring, and temperature regulation. Integration of this platform with theoretical modeling and multiscale biological characterization enables systematic investigation of the mechanisms and potential applications of THz radiation in biological systems.

## Protein Structure and Dynamics

Proteins are the principal effectors of biological activity, supporting catalysis, molecular recognition, transport, and signal transduction through their 3-dimensional structures and dynamic behavior. Rather than adopting a single conformation, they fluctuate among substates whose populations and transition barriers determine active-site geometry, allosteric communication, and molecular interactions [3,86]. Evidence increasingly suggests that THz radiation can redistribute these conformational states and alter functionally relevant dynamics without disrupting the global protein fold.

### Protein conformational rearrangement

Protein structure is determined by sequence-dependent folding and stabilized by intramolecular interactions and the surrounding solvent environment, together providing the structural basis for protein function. As shown in Fig. 4A, owing to its distinctive physical properties, THz radiation can influence protein conformational stability and dynamics by regulating low-frequency molecular vibrations and conformational rearrangements, without inducing substantial macroscopic temperature rise [87]. Unlike classical thermally induced transitions, the effects of THz radiation exhibit pronounced parameter dependence and show reversible and bidirectional response characteristics [27,67,87,88].

**Fig. 4.**
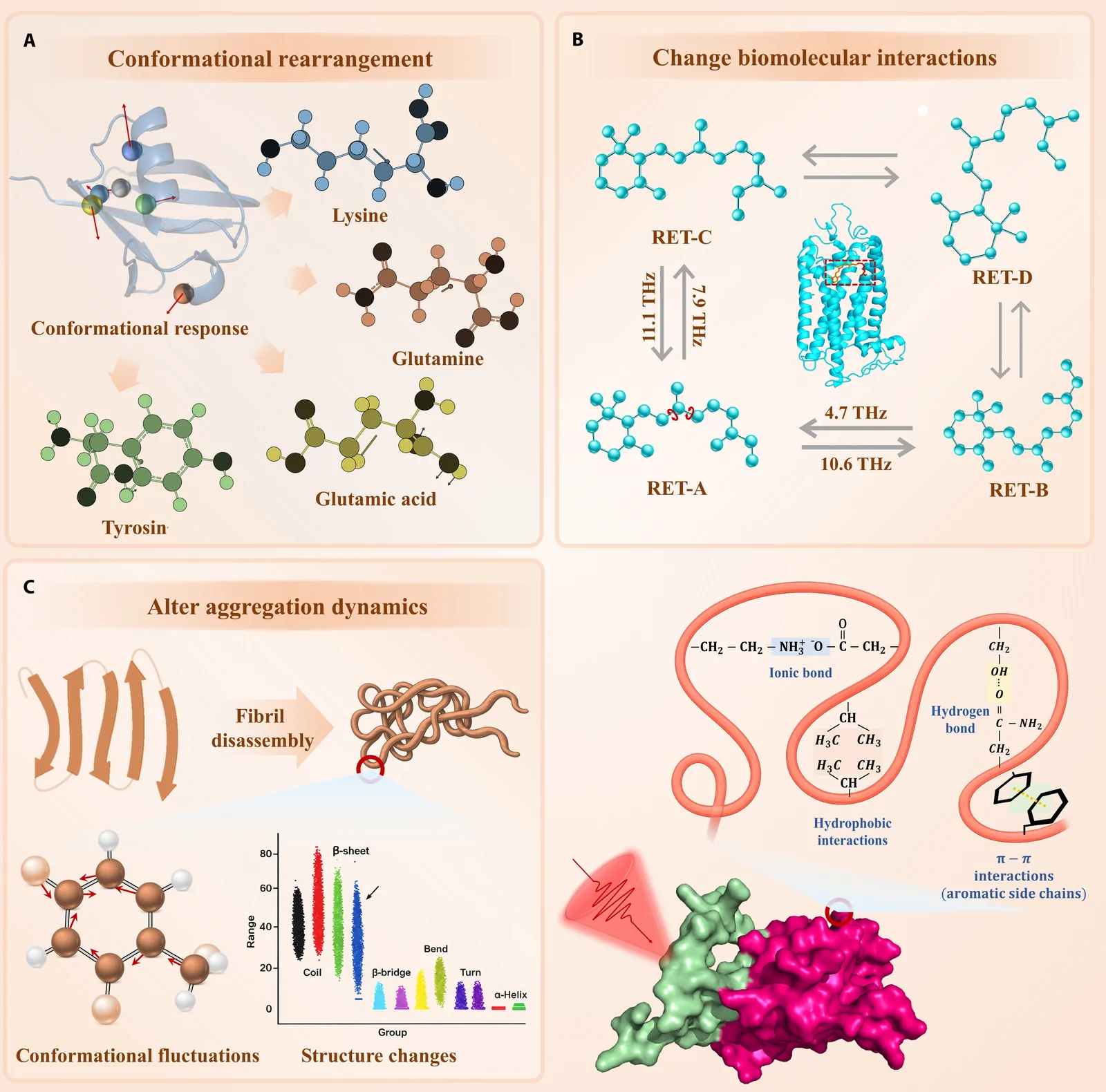
Molecular mechanisms of protein structure and dynamics. (A) Terahertz (THz) fields can couple to low-frequency collective motions and amino acid side-chain vibrations. As illustrated by the distinct conformational responses of lysine, glutamine, glutamic acid, and tyrosine within a similar frequency range, this coupling may differentially modulate local flexibility and conformational transitions, thereby redistributing protein substates without necessarily perturbing the global fold. (B) By selectively amplifying characteristic motional modes, THz radiation reshapes local conformations and noncovalent interaction networks, exemplified by transitions among representative RET-A to RET-D conformers, and ultimately modulating molecular recognition and association–dissociation dynamics. (C) THz irradiation reshapes protein aggregation by altering conformational dynamics and intermolecular interactions, with outcomes ranging from assembly promotion to fibril destabilization depending on exposure conditions and aggregation stage.

Early studies suggested that THz exposure can modulate enzymatic activity and substrate binding by altering the α-helical content and the binding properties of hydrophobic regions [89–91]. Notably, no peptide-chain cleavage or polymerization was observed, and the response was approximately linear with dose over a limited exposure range [91]. Crystallographic simulations have supported this picture in that continuous irradiation at 0.4 THz was reported to enhance local electron density in lysozyme crystals and to compress α-helices along their axes, while leaving the overall B factors and unit-cell parameters largely unchanged. These observations motivated the hypothesis of a Fröhlich-like condensation, in which absorbed energy accumulates in low-frequency vibrational modes to form long-lived coherent oscillations that drive nonthermal structural rearrangements within proteins [87]. In addition, acoustic oscillations in the sub-THz regime can resonantly excite collective protein motions, amplifying intersubunit displacements and conformational fluctuations, and thereby influencing protein compactness and functionally relevant flexibility [27,88,92,93]. This suggests that low-frequency vibrational modes in proteins may serve as important channels for THz energy coupling.

Protein responses also appear to exhibit pronounced spectral selectivity. Jindo et al. [94] showed that irradiation of β2-microglobulin-derived amyloid fibrils at 6.1 μm (~49 THz), corresponding to the amide I band, substantially reduced the β-sheet content and increased the α-helical content, accompanied by marked fibril dissociation. In contrast, irradiation at 6.5 μm (~46 THz; amide II) or 5.0 μm (~60 THz; a weakly absorbing, nonspecific wavelength) produced little detectable conformational change. These findings indicate that protein structural responses can depend on spectral matching to specific vibrational modes, thereby preferentially directing particular conformational transitions.

### Protein–ligand interaction dynamics

There is growing spectroscopic evidence indicating that hydration networks at ligand–receptor interfaces and collective low-frequency motions within binding pockets exhibit characteristic responses in the THz range, raising the possibility of modulating molecular recognition [95]. Early exploratory studies using simplified enzyme–substrate and antigen–antibody systems demonstrated subtle but statistically significant changes in biomolecular interactions, manifesting as reduced catalytic efficiency and decreased stability of preformed complexes [96]. More systematic investigations suggested that THz irradiation can reshape key hydrogen-bond occupancies and binding free energy landscapes via resonance effects, thereby modulating ligand–receptor affinity and specificity [85,95]. For instance, Song et al. [97] reported that 34-THz irradiation enhances relative vibrations at the glutamate-AMPAR-Glu binding site, weakens the hydrogen bond between glutamate and Arg96, and reshapes the binding free-energy landscape. Similarly, exposure to 44.5 THz was shown to weaken critical hydrogen bonding between dopamine and DRD2, resulting in ligand “dedocking” [98]. However, THz effects are not uniformly dissociative. Sun et al. [99] demonstrated that 83-THz irradiation can enhance the binding affinity between DNA and histones, thereby modulating chromatin accessibility and suppressing the expression of genes associated with glycolysis and cell migration, ultimately reducing cancer cell motility. Similarly, in the choline–choline acetyltransferase system, 45.3-THz exposure was found to reduce binding free energy and strengthen interactions with residues such as His324, suggesting that THz irradiation can induce a “stabilizing” rather than “dissociating” effect at defined molecular interfaces [100].

More generally, when the external field frequency matches collective vibrational modes of the internal or interfacial hydration layers in a complex, cooperative motion along the characteristic mode direction can be selectively amplified, thereby reshaping molecular conformations and noncovalent interaction networks, ultimately resulting in changes to the binding free energy landscape and dissociation barriers. In addiction-related pathways, 13.02-THz radiation has been shown to resonant with out-of-plane, symmetric bending and rotational motions of the nicotine molecular ring, disrupting key hydrogen bonds and cation–π interactions with AChBP, with a well-defined field strength threshold (≈0.60 V/nm) and parameter dependence [101]. Similarly, as shown in Fig. 4B, radiation at 8.7 to 8.9 μm (≈34.5 THz) can resonantly activate amino acid side-chain stretching modes, promoting the Fe^3+^–transferrin complex toward dissociation, thereby reducing iron binding capacity and downstream cellular phenotypes [35]. Multiscale simulations in the μ-opioid receptor-fentanyl system further reveal that THz radiation can resonantly couple to characteristic vibrational modes of key reaction coordinates, such as the Asp147 salt bridge and His297 hydrogen bond, introducing orientation-dependent Stark perturbations that dynamically tilt the local potential energy surface and lower dissociation barriers, thereby accelerating ligand unbinding on picosecond time scales [102]. Because absorption and heating may also increase near resonant frequencies, thermal contributions must be carefully distinguished from frequency-selective coupling [83].

### Protein aggregation and disaggregation dynamics

Protein assembly is governed by conformational selection, nucleation, elongation, and disassembly, coordinated through hydrogen bonding, hydrophobic interactions, and electrostatic forces. These processes underpin cytoskeletal organization, functional amyloid formation, and supramolecular machinery assembly. When physiological assembly becomes dysregulated, persistent aggregation can generate stable amyloid fibrils or toxic oligomers associated with neurodegenerative diseases such as Alzheimer’s and Parkinson’s diseases.

As illustrated in Fig. 4C, multiple studies suggest that THz radiation can regulate protein assembly in a frequency-specific and bidirectional manner. Wang et al. [103] found that 3.1 THz promoted early Aβ42 aggregation but inhibited later oligomer-to-protofibril conversion, suggesting that THz exposure may redistribute individual kinetic steps rather than uniformly promote or suppress aggregation. Simulations attributed this stage dependence to changes in secondary structure, hydrophobic surface exposure, and molecular-recognition interfaces. Similarly, 0.1-THz exposure reduced Tau protofibril compactness, weakened hydrogen-bonding and hydrophobic interactions, and decreased aggregation signals in Tau-expressing *Caenorhabditis elegans* [104]. Computational studies further revealed pronounced frequency selectivity. Moreover, a 42.55-THz electric field was predicted to increase the β-structure propensity of Aβ42 monomers, strengthen intermolecular hydrogen bonds and binding in dimers, and mildly stabilize tetrameric protofibrils. By contrast, 20 THz produced limited effects, whereas 34.88 THz weakened interactions between peripheral peptide chains in the protofibril.

Hydration and exposure energy impose additional boundaries on these responses. Continuous 0.6-THz irradiation altered the hydration environment and intermolecular interactions of hydrated α-synuclein and TDP-43 (transactive response DNA-binding protein of 43 kDa) [105] fibrils, whereas near-field exposure under dry conditions weakened fibrillar interactions without demonstrating complete disassembly. Studies by Yamazaki et al. and Hoshina et al. showed that THz irradiation generates transient acoustic waves in aqueous media, disrupting intracellular actin filaments [68,69]. In contrast, low-power quasi-continuous-wave exposure promoted F-actin polymerization and elongation [67]. Intense picosecond THz pulses also induced microtubule depolymerization within minutes, with rates dependent on field strength and spectral properties [106]. Such high-peak-field effects cannot be directly extrapolated to low-power continuous-wave exposure. Likewise, Kawasaki et al. [89] reported that far-infrared free-electron laser irradiation, primarily at 74 and 80 μm, reduced the β-sheet content of calcitonin-derived DFNKF fibrils and progressively disrupted and dispersed fibril bundles as cumulative exposure energy increased.

It is worth noting that oscillating fields may also differ from static fields. Simulations by Kalita et al. [107] showed that oscillating electric fields promoted rapid and persistent Aβ aggregate dissociation, whereas static fields stabilized intermediate peptide-pair structures and produced slower, more reversible disassembly. Protein responses thus depend not only on frequency and field strength but also on the match between field time scales and intrinsic conformational relaxation.

## Nucleic Acid Structure and Dynamics

Nucleic acid function depends not only on sequence but also on dynamic processes such as base pairing, local strand opening, higher-order assembly, and chemical modification, which together underpin the storage, transmission, and expression of genetic information. Base stacking and hydrogen-bond networks stabilize the double helix, whereas the charged phosphate backbone and its hydration shell shape the conformational and dynamical landscape of nucleic acids and provide potential interfaces for coupling with THz fields. Current evidence suggests that THz radiation can influence nucleic acid assembly, strand separation, DNA methylation, and transcriptional activity.

### Nucleic acid assembly and unwinding dynamics

Early spectroscopic studies identified characteristic THz absorption bands in DNA bases and deoxynucleosides, largely associated with collective motions of base-pairing and hydrogen-bond networks [108]. Additionally, the highly charged phosphate backbone further enhances the sensitivity of nucleic acids to external electromagnetic fields, providing a physical basis for THz energy coupling.

In the low-to-mid frequency domain, nucleic acids exhibit sharply distinct conformational responses across frequency bands. Greschner et al. [109] reported that GHz radiation at room temperature can strengthen base pairing and interstrand interactions, promoting self-assembly and structural “repair” in triple-stranded DNA and single-stranded regions of plasmids. In contrast, under high-intensity pulsed irradiation in the ~0.1- to 6-THz range, short DNA strands can experience localized melting or partial unzipping. This reversible conformational response shows a clear dependence on chain length, with minimal evidence of strand breakage or degradation, suggesting that THz fields can modulate nucleic acid conformation and dynamics in a parameter-dependent manner without inducing appreciable damage. Consistent with this, cellular experiments have demonstrated that THz exposure at 200 μm (≈1.5 THz) can substantially reduce DNA synthesis rates in mammalian cells [110], further supporting a regulatory role of THz radiation in nucleic acid processes.

As the frequency increases toward the extended THz regime, the effects become more closely associated with specific collective vibrational modes. Continuous-wave irradiation at 35.2 THz has been shown to resonantly enhance pyrimidine vibrational modes and, under nonthermal conditions, induce hydrogen-bond rupture, leading to DNA duplex unwinding at temperatures ~10 °C below the melting point, thereby facilitating the reconfiguration of misfolded structures [111]. Notably, the assembly fidelity of DNA origami structures was improved to >80%, representing an approximately 3-fold enhancement over conventional thermal annealing (Fig. 5B). Complementary simulations indicate that 44-THz irradiation can resonantly excite in-plane stretching modes of purine bases, weakening hydrogen bonding and substantially accelerating strand separation, resulting in a ~20-fold increase in the unwinding rate and a substantial decrease in the effective unwinding temperature [112]. Furthermore, theoretical work by Udoisoh and Olajide [113] proposed that THz fields can dynamically modulate spin-dependent transport in chiral nucleic acids by reshaping electronic bands and Berry curvature through a Floquet-chirality-induced spin selectivity mechanism. These findings suggest that THz fields may influence not only nucleic acid conformation but also electronic and spin dynamics.

**Fig. 5.**
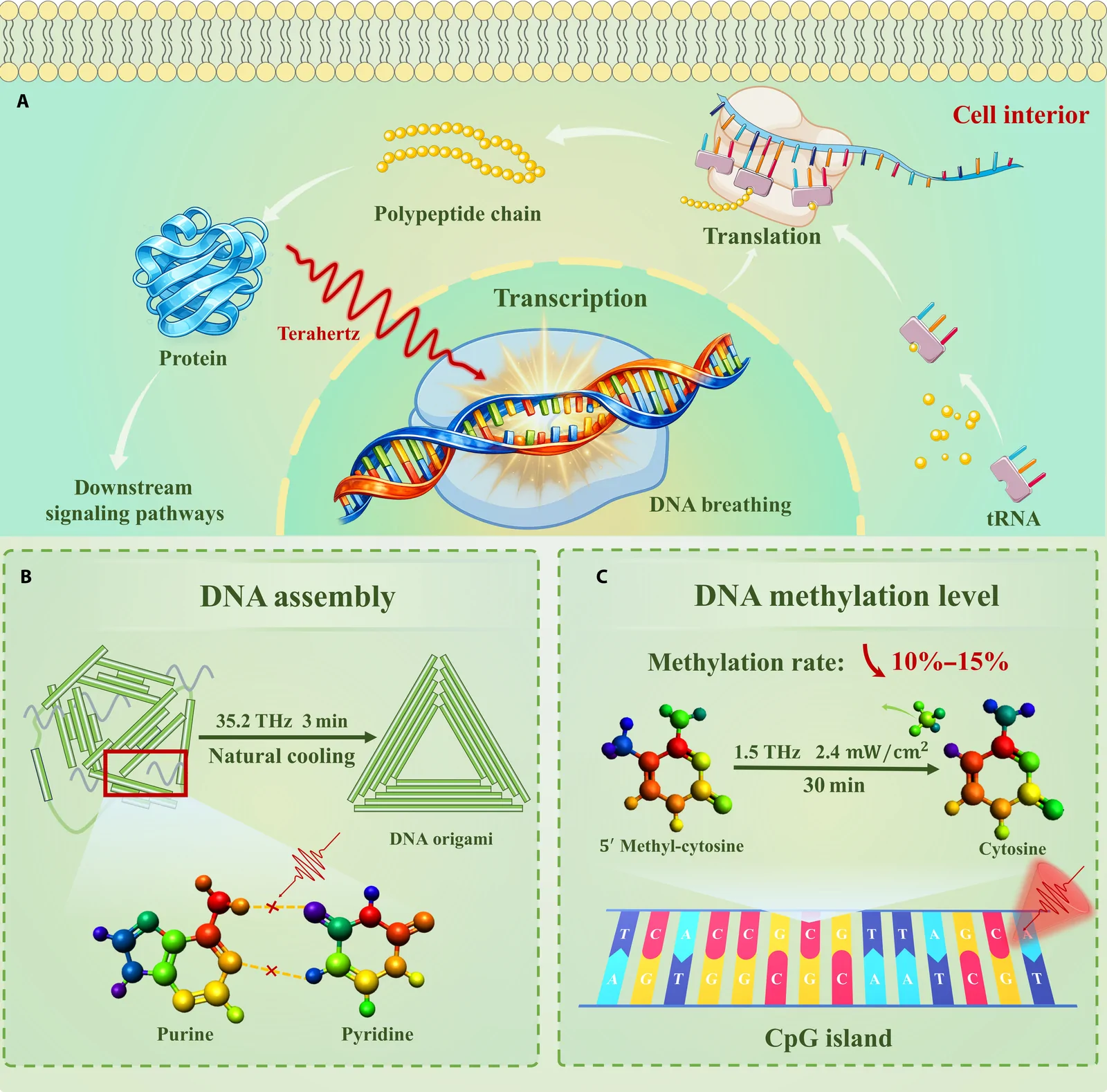
Molecular mechanisms of nucleic acid structure and dynamics. (A) Terahertz (THz) fields may couple to collective motions of nucleic acids and promote local DNA-breathing dynamics. By altering promoter accessibility, these changes may further influence transcription and translation, protein synthesis, and downstream signaling pathways. (B) THz irradiation can resonantly enhance nucleotide-base-associated vibrational modes and weaken interstrand hydrogen bonding, thereby promoting the rearrangement of misassembled DNA structures and improving DNA origami assembly. (C) Low-power irradiation near 1.5 THz has been associated with a reduction in DNA methylation, illustrated by the loss of methylation-associated groups from 5-methylcytosine and an approximately 10% to 15% decrease in global methylation.

### DNA methylation status

Epigenetic modifications, particularly DNA methylation, play central roles in gene regulation and genome stability, and their dysregulation is tightly linked to tumorigenesis [114,115]. Spectroscopic work by Cheon et al. [116] suggested that DNA isolated from multiple hematological malignancy cell lines (including T-cell lymphoma, B-cell lymphoma, and acute myeloid leukemia) exhibits a characteristic absorption feature near ~1.7 THz that correlates with methylation status, with peak intensity positively associated with global methylation level, raising the possibility that THz fields could modulate DNA methylation via resonance effects.

To test whether this spectral feature could be functionally targeted, the authors exposed cell-free DNA to irradiation centered at approximately 1.5 THz with a power density of 2.4 mW/cm^2^. Variable reductions in methylation were reported in artificially methylated DNA and DNA isolated from several tumor cell lines [117]. Agreement between THz time-domain spectroscopy and enzyme-linked immunosorbent assay, together with temperature monitoring and damage assessment, led the authors to attribute the effect primarily to disruption of methylation-associated bonds rather than bulk heating. The approach was subsequently extended to melanoma cells (SK-MEL-3). Under low-power, short-duration exposure (40 μW, 30 min), global methylation was reduced by ~10% to 15% (Fig. 5C), with only mild DNA damage (~5 to 8 abasic sites per 10^5^ bp), substantially below an infrared-laser control (160 mW, 800 nm). These findings suggest that within an appropriate dosimetric window, THz irradiation may enable nonthermal, controllable demethylation and point toward potential applications in precision epigenetic modulation.

More recently, the same group reported that THz exposure at 1.6 THz and 14.7 $\mathrm{mW}/{\mathrm{cm}}^{2}$ modulated gene expression in cancer cells. A 30-min treatment reduced global DNA methylation by approximately 19% in a power-dependent manner, while abasic-site levels remained below 1 per 10^5^ bp. Microarray analysis revealed marked remodeling of pathways associated with stress, inflammation, and apoptosis. Notably, FOS, JUN, and CXCL8 were down-regulated by more than 4-fold within 24 h postirradiation. On this basis, the authors proposed that THz irradiation may exert antitumor effects at the epigenetic level by modulating the activator protein 1 transcription factor complex and the methylation state of its target genes [118].

### DNA breathing dynamics

Under physiological conditions, thermal fluctuations cause spontaneous and transient local opening of the DNA double helix, generating short single-stranded bubbles commonly referred to as DNA breathing [119,120]. Such transient openings alter local accessibility and mechanical flexibility, thereby facilitating the binding of transcription factors and regulatory proteins.

In 2010, Alexandrov et al. [121] used an extended Peyrard–Bishop–Dauxois model to investigate the physical basis of THz-driven DNA breathing. Simulations at 2.0 to 2.52 THz with a driving amplitude of 144 pN showed that frequencies near 2.5 THz shifted the system from uniform to staggered oscillations and induced period-doubling instability (Fig. 5A). Above a threshold driving amplitude, localized DNA bubbles emerged together with subharmonic responses and spatially dimerized oscillations. These results suggested that THz fields can resonantly excite hydrogen-bond motions, alter interstrand interactions and stack energies, and promote transient opening of promoter regions without requiring substantial bulk heating.

Subsequent cell-based studies provided indirect experimental support for this mechanism [122]. Under irradiation with a center frequency of ~10 THz, prolonged exposure (12 h) markedly up-regulated adipogenic differentiation genes in mouse mesenchymal stem cells (PPARG, adiponectin, GLUT4, and FABP4), consistent with enhanced adipogenic commitment. By contrast, short exposure (2 h) primarily affected pluripotency-associated genes, suggesting a reversible modulation of early stem-cell states. Complementary molecular simulations further indicated that specific promoter regions (for example, Gem and Slco4a1) exhibit elevated local opening–closing activity under irradiation, leading to the hypothesis that promoter-specific breathing propensities may contribute to gene-selective transcriptional responses.

Independent experiments by Bock et al. [123] further examined mouse mesenchymal stem cells exposed to approximately 10-THz radiation at an average power density of about 1 mW/cm^2^ for 2 to 9 h under strict temperature control. Although no overt membrane damage was detected, longer exposures of 6 to 9 h produced lipid accumulation and adipocyte-like phenotypes. Transcriptome profiling again revealed gene-specific shifts (~6% up-regulated, ~5% down-regulated), enriched in adipogenic pathways. Combined with EPBD-Langevin simulations, the author noted that the PPARG promoter preferentially supports stable or highly occupied local openings, in contrast to the PPARA promoter. These findings support a model in which THz irradiation selectively enhances transcription by modulating local DNA-breathing dynamics through resonant interactions under nonthermal conditions.

## Ion Channels and Ionic Dynamics

Ion channels serve as pivotal molecular nodes for cellular excitability, signal transduction, and homeostasis [124,125]. Their selective permeability is governed primarily by the selectivity filter (SF) and the adjacent amino acid residues, where charged and polar functional groups establish distinct coordination environments [126–128]. During permeation, ions typically undergo successive steps of dehydration, coordination, and rehydration, enabling efficient selective transport across the membrane [129–132]. As shown in Table 2, THz radiation has been proposed to perturb this process by resonantly coupling to intrinsic vibrations of functional groups within the SF, thereby reshaping permeation pathways and kinetics and ultimately modulating ionic flux. This mechanism provides a promising avenue for exploring THz-based modulation of ion channels.

**Table 2.** Molecular mechanisms underlying ion channel regulation by THz radiation

Type	Object	Simulation parameters	Coupling target	Molecular response	Refs.
K^+^	K_V_1.2 voltage-gated channel	1.4, 2.2, 2.9, 5.0, 10.8, 75 THz; 0.2–0.8 V/nm	Filter-confined K^+^ at low frequencies; hydration-water hydrogen bonds and ion-occupancy networks at 10.8 THz	1.4–2.9 THz enhances axial K^+^ transport by lowering interstice barriers, and 10.8 THz disrupts conductive ion occupancy across S0–S4.	[137]
		4, 10, 15, 20 THz; 0.4 V/nm; axial field	Thr374 and its water-mediated hydrogen bonds at the filter entrance; K^+^ coordination sites	The field reduces hydrogen bonding between Thr374 and water, promotes K^+^ dehydration and reorganizes ion–water occupancy.	[149]
		6, 15, 25 THz; 0.4 V/nm; axial field	Confined water in the central cavity and the hydration shell at the filter entrance	At 15 THz, enhanced water dynamics accelerate cavity-to-filter K^+^ exchange and lower the dehydration barrier.	[189]
		36.75, 37.06, 37.68, 38.20 THz; 0.4 V/nm	Gly376 backbone carbonyls and tetrameric filter symmetry	36.75 THz strengthens correlated carbonyl vibrations and increases backbone dihedral fluctuations, disrupting filter symmetry and ion coordination.	[140]
	KcsA channel	51.87 THz; 0.4 V/nm; axial field	SF carbonyls, particularly sites near Tyr78	Resonant coupling to C=O stretching enhances coordinated motion between carbonyl oxygens and permeating K^+^ ions, stabilizes multi-ion occupancy and lowers migration barriers.	[139]
		51.87 THz; 0.4 V/nm; field angles of 0°, 30°, 45°, 60°, 90°	Gly77 backbone carbonyls, carbonyl orientation and ion occupancy	Axial fields preserve carbonyl coordination and favor K^+^ migration, whereas transverse fields increase Gly77 dihedral deflection and destabilize the filter.	[151]
	Simplified KcsA SF model	53.00, 53.60, 53.75, 54.00 THz; ~2.5 V/nm	Carbonyl array within the model pore	53.60–53.75-THz irradiation induces coherent carbonyl reorientation and generates transient asymmetric fields that favor axial K^+^ migration.	[136]
Na^+^	Artificial selective nanopore	48.2, 36 THz; ~1.0 V/nm; field along the membrane normal	Glutamate residues and the Na^+^ hydration shell	At 48.2 THz, excitation of –COO^−^ vibrations transfers energy to Na^+^ through hydrogen-bonded water and weakens hydration–water interactions with the filter, reducing ion confinement.	[190]
	Nav1.5 voltage-gated channel	2.5, 10, 48.6, 75 THz; 0.2–0.8 V/nm	Filter-confined Na^+^ at 2.5 THz; DEKA carboxylates at 48.6 THz	At 2.5 THz, direct excitation accelerates axial Na^+^ motion, whereas 48.6-THz carboxylate vibrations modulate ion confinement through the hydration network.	[137]
Ca^2+^	CaVAb voltage-gated channel	42.55, 51.61 THz; ~0.6 V/nm; axial field	Symmetric carboxylate stretching at 42.55 THz; backbone carbonyl vibration at 51.61 THz	Excited –COO^−^ or C=O groups couple to the Ca^2+^ hydration shell through hydrogen bonding, thereby modulating Ca^2+^ dynamics.	[133]
	Simplified channel-barrier model with CaV structure	1-THz unipolar pulse; ~3 × 10^8^–6 × 10^8^ V/nm; 2-fs time step	Ca^2+^ confined within the channel potential	Resonant driving enhances axial Ca^2+^ motion and accelerates barrier crossing.	[152]
	CaV1.2 SF models	42.5 THz	Asp/Glu carboxylates and the Ca^2+^–water coordination network	Enhanced –COO^−^ stretching reorganizes Ca^2+^ hydration and lowers the permeation barrier.	[36]
	Double-Ca^2+^ system in the CaVAb selectivity filter	1.65, 2.84, 10 THz; 0.3 V/nm for calculations; 3 V/nm for temperature analysis	Collective motions of 2 adjacent confined Ca^2+^ ions	Ca^2+^ ions move in phase at 1.65 THz and out of phase at 2.84 THz; amplifying the in-phase mode promotes forward migration through concerted electrostatic repulsion.	[153]

THZ, terahertz; SF, selectivity filter

### Ion channel gating dynamics

An expanding literature suggests that THz fields can resonantly couple to polar functional groups in the SF, remodel the free-energy landscape of transmembrane transport, and alter permeability. In potassium channels, multiple studies report that THz irradiation enhances K^+^ currents and increases channel conductance by resonantly engaging carbonyl (–C=O) groups enriched within the SF [133–140]. Biological experiments have further linked such modulation to nervous system phenotypes, including suppression of visually evoked escape responses in zebrafish [141] and reduced excitability of anterior cingulate cortex pyramidal neurons [142]. Along similar lines, Wei et al. [143] reported that 0.1- to 3-THz exposure elevates intracellular Ca^2+^ levels in sperm cells without detectable DNA damage, enhancing motility. Li et al. [144] observed that 2.52-THz irradiation promotes angiogenesis in human umbilical vein endothelial cells by increasing permeability of voltage-gated calcium channels.

A key point is that resonant coupling is often accompanied by a coordinated response of the hydration hydrogen-bond network. Excitation of group vibrations can simultaneously alter hydrogen-bond strength and orientational correlations between SF functional groups and surrounding water molecules, enabling indirect control of permeation through hydration-layer reorganization [138,145]. In ${\mathrm{Ca}}_{v}$ channels, for instance, Li et al. [133] found that Ca^2+^ traverses the SF with minimal direct coordination to functional groups, instead relying on water-mediated hydrogen-bond pathways to achieve effective conduction. In this setting, selective excitation of carboxylate (–COO^−^) or carbonyl (–C=O) modes could modulate the functional-group-hydration hydrogen-bond network (either strengthening or weakening it), lowering the barrier for ion crossing and increasing Ca^2+^ influx (Fig. 6B). Notably, under a forced-vibration mechanism, even when the external field is primarily tuned to C=O resonances, COO^−^ groups can still contribute to enhanced permeation. Song et al. [137] further reported direction selectivity, with pronounced enhancement when the field is aligned with the channel axis (Z axis) and minimal or no effect when polarized perpendicular to the axis (X/Y). Together, these findings support a multistep energy-transfer mechanism in which functional-group vibrations reorganize the surrounding water network and thereby reshape the ionic free-energy landscape.

**Fig. 6.**
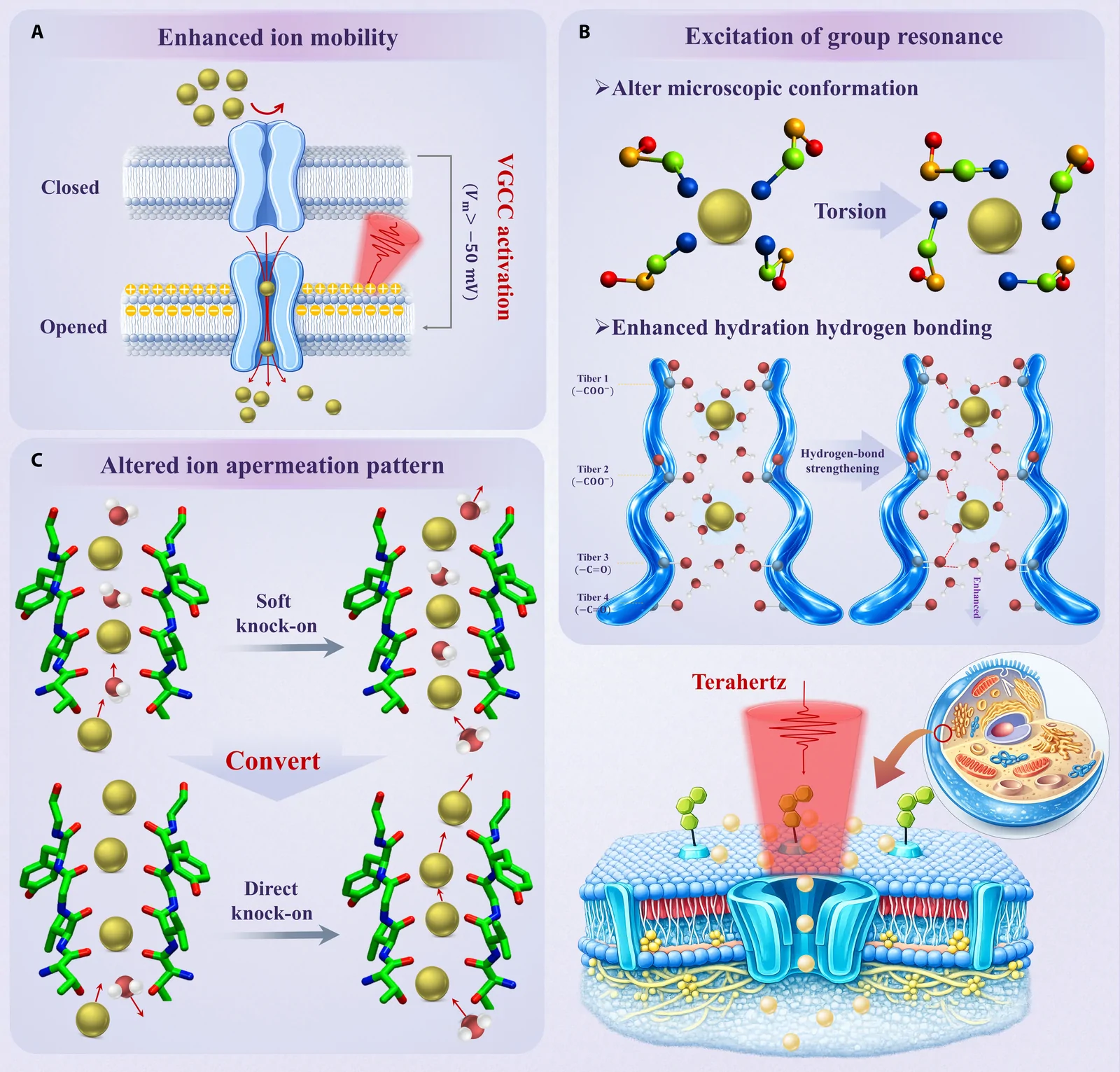
Molecular mechanisms of ion channels and ionic dynamics. (A) Terahertz (THz) radiation activates voltage-gated ion channels through electromagnetic interactions, enhancing transmembrane ion flux, as exemplified by calcium channels. (B) THz radiation can modulate the local conformation of the selectivity filter (SF) and the hydrogen-bond network of its hydration layer via resonance effects, altering ion-channel permeability. (C) THz radiation alters the stability of the hydration layer at the SF entrance, the orientation of water dipoles, and the hydrogen-bond network, thereby modulating ion permeation patterns and influencing transmembrane ion transport efficiency.

Simulations additionally indicate that SF responses vary with the frequency and polarization of the applied field, resulting in either enhanced or suppressed conductance. Zhao et al. [140] reported that 36.75-THz irradiation excites a carbonyl resonance at GLY376, inducing torsion of the backbone dihedral angle (N-Cα-C-O) and distorting the upper segment (S0-S2), thereby amplifying structural asymmetry within the filter (Fig. 6B). As the carbonyl flip angle and radial displacement increase, the occupancy distribution of K^+^ within the SF shifts, the effective barrier decreases and permeation is promoted. By contrast, in the same system, irradiation at 53 and 54 THz, corresponding to nonresonant or orthogonally polarized carbonyl modes, produced no appreciable enhancement. Similarly, Wu et al. [136] found that 53.6-THz exposure can resonantly excite in-plane carbonyl stretching, strengthening carbonyl flipping and radial displacement and inducing SF asymmetry to facilitate K^+^ passage. Conversely, Sajadi et al. [146] reported that lower-frequency radiation can stabilize the KcsA filter by suppressing carbonyl motion, increasing rigidity and constraining cooperative ionic motion. Notably, when the frequency reaches ≥2.5 GHz, cooperative transport nearly vanishes, suggesting that external fields may also push the system toward a more rigid, lower-permeability state.

### Ion permeation dynamics

Ion migration through the SF often proceeds via a highly ordered “knock-on” mechanism, reflecting a coupled process of mutual repulsion and replacement between ions and water molecules within a confined geometry [131,147,148]. Depending on the ion–water arrangement, permeation can be categorized into direct knock-on, soft knock-on, or mixed modes. Because these modes differ in dehydration extent, local barriers and flux characteristics, switching between them can substantially change channel permeability.

Consistent with this framework, multiple studies suggest that THz fields can alter the statistical weighting of permeation modes by modulating hydration stability at the SF entrance, local dipole orientation, and the hydrogen-bond network. Ding et al. [149] reported in simulations that 15-THz irradiation does not induce pronounced resonance or major conformational rearrangement within the SF but deepens the potential well at the Scav site and weakens hydrogen-bond constraints between entrance water molecules and carbonyls, destabilizing the local hydration shell. This facilitates ion entry into the filter in a partially dehydrated state, enhancing direct knock-on conduction (Fig. 6C). Wang et al. [139] reported consistent conclusions in the KcsA system, where 51.87-THz irradiation resonantly couples to SF carbonyls, stabilizes filter structure, and lowers the transmission barrier, markedly increasing the fraction of direct knock-on events and thereby boosting conduction efficiency. In contrast, Adiban et al. [150] found that low-frequency THz exposure can deepen potential wells at specific sites in Ca^2+^ channels, strengthening ion trapping and restricting migration pathways, resulting in reduced permeability, again emphasizing parameter dependence and potentially opposite outcomes.

Notably, the incidence direction (or polarization relative to the channel axis) may be a decisive variable in permeation-mode bias. Simulations indicate that when a 51.87-THz field is aligned with the direction of K^+^ flow (θ = 0°), the proportion of direct knock-on transport increases and the permeation rate rises to ~1.8× that of the field-free condition [151]. When the field is perpendicular to the flow (θ = 90°), soft knock-on becomes more prevalent and flux decreases. Importantly, this regulation does not require strong resonance or gross structural distortion. Rather, it is attributed to anisotropic modulation of local dipoles and hydrogen-bond networks by the THz field. At θ = 90°, orientational fluctuations of the GLY77 φ dihedral angle are enhanced, locally destabilizing the SF and promoting K^+^ retention in S3-S2 or S4-S3 configurations, consistent with a shift toward soft knock-on transport.

### Ion migration dynamics

Beyond the mechanisms discussed above, Guo et al. [152] proposed an alternative coupling pathway from the perspective of electromagnetic response. Using Brownian dynamics simulations, they reconstructed the 3-dimensional potential energy landscape of Ca^2+^ ions within voltage-gated calcium channels and further estimated the spontaneous electromagnetic radiation associated with ionic motion. Their results showed that a single Ca^2+^ ion can generate a characteristic radiation frequency of ~16.9 THz. As the number of ions increases, the radiation intensity is markedly enhanced while the central frequency largely unchanged. This observation suggests that interionic interactions, such as Coulomb-driven acceleration and deceleration, may cooperatively amplify the radiation process. Upon application of an external THz field, the spectral peak of the spontaneous radiation is further increased, leading to the proposal that THz irradiation may facilitate Ca^2+^ permeation by enhancing the electromagnetic response of ions and lowering the effective energy barriers for transport. This work also highlights that the aqueous environment within the channel differs substantially from bulk water. In the confined neck region, negatively charged residues reshape the polarization state of water molecules and reduce the effective dielectric constant, thereby promoting transient ion trapping within local potential wells instead of continuous diffusion and providing a physical context for the coupling among the energy landscape, hydration dynamics, and ion transport. Building on this framework, Zhang et al. [153] further demonstrated that the collective motion of confined Ca^2+^ ions exhibits not only classical vibrational characteristics but also discrete quantum eigenstates, with transition frequencies closely matching the observed vibrational modes. These findings suggest that the response to external fields may involve selective excitation of collective ionic states and providing a more refined physical perspective for understanding THz-modulated high-flux ion transport.

In a related but distinct stimulation regime, Bo et al. [154] used particle-based simulations to show that nanosecond pulsed electric fields can transiently alter membrane potential and activate low-threshold voltage-gated calcium channels, producing substantial Ca^2+^ influx on ultrashort time scales (Fig. 6A). Cellular modeling further suggested that a unipolar pulsed exposure at 0.5 THz with a pulse width of 1.2 ns (field strength 5 × 10^7^ V m^−1^) can markedly elevate the membrane potential of rat striatal cells and induce hydrophilic pore formation at ~0.94 ns, driving a transient surge in transmembrane fluxes of Na^+^, K^+^, Cl^−^, and Ca^2+^, followed by exponential decay. Concurrently, aberrant membrane-potential excursions can transiently activate Na^+^/K^+^-adenosine triphosphatase, promoting Na^+^ extrusion and K^+^ influx and leaving a nanosecond-scale kinetic “tail” after the stimulus ceases. Notably, similar conclusions were retained across a range of frequencies (0.1 to 1.2 THz), providing a theoretical basis for interpreting intracellular information transfer and regulation under THz stimulation.

## Lipid and Membrane Structure and Dynamics

Biological membranes form the key interface and selective barrier between the intracellular and extracellular environments, underpinning a wide range of functions including cytoskeletal organization, homeostatic regulation, and metabolism. As a primary sensor and responder to external stimuli, the membrane is not only a major target of environmental perturbations but also a critical mediator that transduces extracellular cues into intracellular signals. Accordingly, a mechanistic understanding of electromagnetic interactions between THz radiation and membrane systems is essential for clarifying the molecular basis of THz bioeffects and for advancing translational applications.

### Membrane structure and permeability

Across diverse model systems, the plasma membrane was among the earliest targets studied and remains one of the most intensively investigated. Early reports converged on the notion that THz irradiation can perturb membrane organization, increase permeability, and alter electrophysiological function, typically with strong dose- and frequency dependence. Olshevskaya et al. [155] found that exposure to ~3.65 THz (7 to 418 μm) for 40 to 50 h produced heterogeneous surface structures in approximately 12% to 15% of neurons of *Lymnaea stagnalis*, which were accompanied by randomly oriented bud-like protrusions. Continued exposure led to thickening of these protrusions and impaired cell–cell connectivity. Moreover, at higher power (≥30 $\mathrm{mW}/m^{2}$), only 1 min of irradiation was sufficient to induce disordered membrane morphology, membrane-potential disruption and intracellular structural perturbations, whereas at lower power (1 to 10 $\mathrm{mW}/m^{2}$) the phenotype manifested as time-accumulated electrophysiological dysfunction. Related effects have been observed in microbial and erythrocyte models. In *Escherichia coli*, Ivanova et al. [156] reported that 4-THz irradiation caused a dehydration-like morphology together with cytoplasmic leakage, membrane disturbance, separation of inner and outer membranes, and cell-wall defects, with progressively more pronounced membrane damage upon longer exposure. In rat erythrocytes, Kovalenko et al. [157] showed that 30.61 THz (9.8 μm) accelerated hemolysis and increased primary lipid-peroxidation products, whereas 4.25 THz at a comparable power density produced no significant effect, supporting frequency dependence of THz membrane responses. Multiple studies using hemoglobin release and related readouts have further supported the conclusion that THz exposure increases blood-cell membrane permeability [40,158,159].

Beyond overt disruption, reversible and nominally nonthermal modulation of membrane permeability and dynamics has also been demonstrated. Perera et al. [160] showed that irradiation across 0.3 to 19.5 THz can increase membrane permeability of PC12 chromaffin-like cells under nonthermal conditions, facilitating cellular uptake of nanoparticles of 23.5 nm (single particles) and 63.9 nm (aggregates), consistent with measurable membrane deformation and remodeling. Further, using synchrotron-based FTIR (SS-FTIR), increased lipid and cholesterol content after exposure, together with changes in amide I/II bands and ${\mathrm{CH}}_{2}/{\mathrm{CH}}_{3}$ vibrations, findings pointing to coordinated adjustments of membrane composition and membrane-associated protein structure [161]. In line with these observations, Pikov et al. [162] suggested that even at substantially lower frequencies (0.06 THz), external fields may modulate neuronal excitability by altering the electrical properties of the plasma membrane.

Importantly, THz effects are not confined to the plasma membrane. In vitro experiments by Borovkova et al. [163] indicated that continuous-wave irradiation at 0.12 to 0.18 THz (3.2 $\mathrm{mW}/{\mathrm{cm}}^{2}$) can depolarize the mitochondrial membrane potential, with clear dose-dependent cytotoxicity. In SH-SY5Y neuroblastoma-like cells, Lei et al. [164] observed that 0.1-THz exposure (33 $\mathrm{mW}/{\mathrm{cm}}^{2}$, 30 min) increased mitochondrial aspect ratio, expanded the fractional area of the intermembrane space, reduced matrix volume, and elevated membrane potential. These results suggest that membrane electrochemical state may represent a sensitive node in THz responses. In addition, Ramundo-Orlando et al. [165] reported that 130-GHz pulsed irradiation substantially increased the permeability of cationic liposomes loaded with carbonic anhydrase, particularly under 7-Hz low-frequency modulation, which raised the reaction rate from 0.0026 to 0.0045 (ΔA / min).

At the mechanistic level, existing studies can be broadly categorized into 2 parallel, yet potentially coupled, routes. One route invokes modulation of water–lipid interfacial dynamics, altering membrane order and diffusion/permeation behavior. The second route posits resonant coupling to lipid (or headgroup) vibrational modes, driving conformational relaxation and reshaping energetic barriers. Hoshina [166] reported that 0.10- and 0.29-THz exposure increased lipid diffusion coefficients, while Laurdan imaging indicated decreased membrane order, and the authors proposed that THz fields may perturb water–lipid interactions and promote an “ordered-to-disordered” transition (Fig. 7A). Molecular dynamics simulations by Zhang et al. [167] show that dipalmitoylphosphatidylcholine bilayers exhibit a pronounced peak in water flux ~10 THz, accompanied by marked changes in membrane thickness and conformational parameters, displaying an “on-off” gating behavior. The correspondence between frequency and the intrinsic vibrational modes of dipalmitoylphosphatidylcholine suggests that THz irradiation may induce membrane relaxation and lower transmembrane energy barriers via resonant coupling, thereby modulating water permeability. More recent studies indicate that THz fields can promote ion adsorption at the membrane surface by disrupting ion hydration shells, thereby leading to asymmetric membrane restructuring and giving rise to an abnormal reverse osmosis phenomenon [168]. In a related simulation, Hao et al. [169] reported that 42.55-THz irradiation resonantly couples to vibrational modes associated with –COO^−^ and −CH, inducing rotation of negatively charged phospholipid headgroups and redistributing charge within the bilayer. This modulated interactions between synaptotagmin-1 and phospholipids, reduced binding energy and potentially altered neurotransmitter release. Experimentally, Hu et al. [170] found that continuous-wave 0.1-THz exposure enhanced endocytic activity in both mouse hippocampal neurons (HT22) and PC12 cells and promoted intracellular accumulation of small-molecule dyes, suggesting that altered permeability may involve both passive diffusion and active transport processes.

**Fig. 7.**
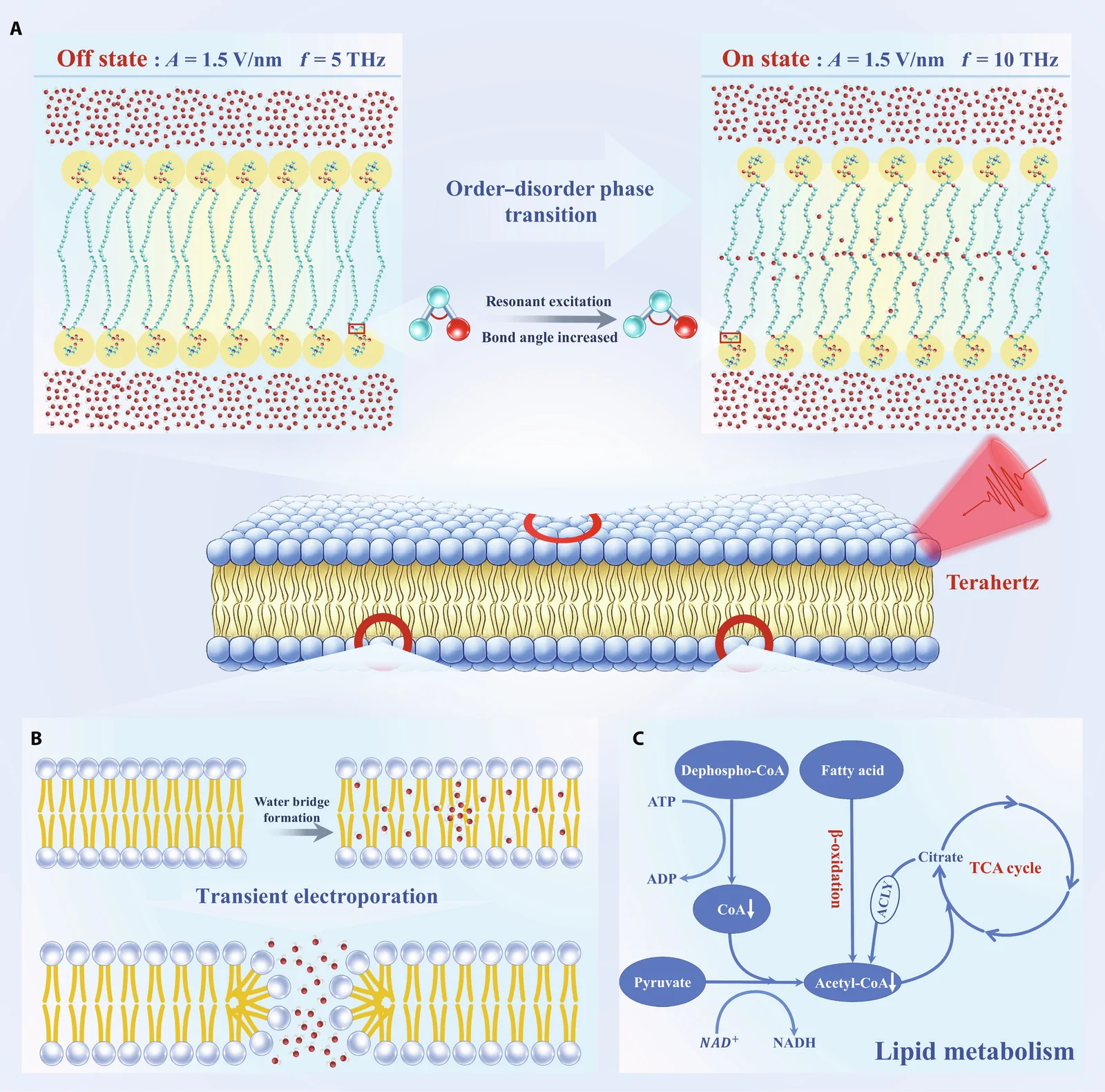
Molecular mechanisms by which terahertz (THz) radiation modulates membrane properties and lipid metabolism. (A) THz radiation induces membrane structural relaxation and reshapes transmembrane energy barriers via resonance effects, thereby altering membrane permeability. (B) THz radiation promotes water-bridge formation and transient pore formation, thereby temporarily increasing membrane permeability. (C) THz radiation modulates lipid metabolism, indirectly reshaping membrane physicochemical properties and function. CoA, coenzyme A; ATP, adenosine triphosphate; ADP, adenosine diphosphate; NAD^+^, nicotinamide adenine dinucleotide (oxidized form); NADH, nicotinamide adenine dinucleotide (reduced form); ACLY, ATP citrate lyase; TCA, tricarboxylic acid.

### Lipid metabolism and membrane properties

In addition to directly perturbing membrane structure and permeability, THz exposure may indirectly remodel membrane physicochemical properties and functional states by regulating lipid-metabolic pathways (Fig. 7C). Because lipids are core structural components of membranes, changes in lipid metabolism can directly affect membrane fluidity, curvature, thickness, and signaling competence. This indirect route therefore provides an important complement for interpreting THz bioeffects.

At the tissue level, Zhang et al. [171] used metabolomics to show that 3-THz irradiation markedly alters lipid distributions in skin, particularly the relative abundances of triacylglycerols, glycerophospholipids, and sphingolipids, implicating key pathways such as fatty acid synthesis and glycerophospholipid metabolism. They proposed that these changes may influence membrane-lipid composition and lipid-raft organization, thereby modulating immune-cell receptor function and signaling to produce downstream immunoregulatory effects. At the cellular level, Butikova et al. [172] examined 2.3-THz exposure in human melanoma cells (SK-MEL-28). They reported substantial perturbations in purine and pyrimidine metabolism, including increased levels of adenosine triphosphate and adenine degradation products (such as xanthine and hypoxanthine), increased uridine and pseudouridine, and decreased uridine monophosphate and cytidine diphosphate, consistent with disrupted energy metabolism and nucleotide homeostasis. Notably, ceramide levels increased, while coenzyme A and its precursor dephospho-coenzyme A decreased, indicating breakdown of lipid-metabolic homeostasis. Combined with gene-network analyses, the authors suggested that THz irradiation may affect mitochondrial membrane-associated proteins (including adenosine triphosphate synthase and nicotinamide adenine dinucleotide [reduced] dehydrogenase), thereby altering enzymatic activity in energy metabolism and producing systemic remodeling of membrane function and cellular metabolic profiles.

### Membrane electroporation and nanopore formation

Electric-field-induced membrane permeabilization is a classical physical strategy to control transmembrane transport, widely used in gene transfection, drug delivery, and cell engineering. Conventional electroporation relies on microsecond-to-millisecond high-intensity pulses. When pulse durations shrink to picosecond or subnanosecond regimes, however, the field effects may extend beyond the plasma membrane to intracellular organelles and cytoskeletal structures, with more complex biological consequences. In recent years, several studies have reported that THz irradiation can facilitate entry of macromolecules that normally do not readily cross membranes (for example, dyes and hemoglobin) without detectable bulk temperature changes and with partial reversibility [173]. This observation supports the hypothesis that THz fields drive localized hydrophilic rearrangements, generating transient self-sealing nanopores and increasing the probability of transmembrane passage for hydrophilic solutes (Fig. 7B).

Molecular-dynamics simulations have provided a mechanistic microphysical picture. Tang et al. [174] showed that a unipolar trapezoidal THz pulse (0.5 THz, 0.65 V/nm) rapidly increases the alignment of surface-water dipoles along the field direction, driving interfacial water protrusions that extend into the bilayer and ultimately form a stable water-bridge structure, effectively a nanoscale electropore. Under the same field strength, a periodically reversing sinusoidal waveform failed to trigger pore formation, indicating strong waveform sensitivity of electroporation under nonextreme fields. Frequency scans further suggested that within 0.1 to 0.9 THz, pore-formation time generally increases with frequency, yet an anomalous acceleration emerges around 0.4 to 0.5 THz. This is consistent with a resonance window of the water hydrogen-bond network in which the external field accelerates bond formation and rupture dynamics, thereby speeding pore formation. In agreement, under bipolar picosecond pulse trains, interfacial water molecules continuously flip and reorient with field reversal, preventing sustained directed motion away from the phospholipid–water interface, an effect analogous to bipolar cancellation. Importantly, pore formation occurs predominantly within the lipid bilayer rather than through channel proteins themselves. The presence of membrane proteins can nevertheless shift the mean pore-formation time, with system-size dependence in simulations.

Complementary experimental evidence has been reported at subnanosecond time scales. Vernier et al. [175] showed that a single high-field pulse (≤500 ps; ~19 MV/m) can depolarize rat hippocampal neurons and trigger intracellular Ca^2+^ transients that persist for >100 ns in a dose-dependent manner, consistent with rapid transient electropermeabilization. Corresponding molecular-dynamics studies indicate that, on subnanosecond time scales, strong electric fields drive interfacial water into the bilayer to form stable hydrophilic channels. Even under rapidly oscillating THz-range fields, water dipoles can flip synchronously within picoseconds and maintain transmembrane water bridges, leading to pronounced hydrophilization and local structural disorder over tens of picoseconds.

Furthermore, Bo et al. [176] theoretically analyzed how pore formation influences ionic transport and membrane conductance under THz electric fields. Their results suggested that picosecond Gaussian THz pulses increase membrane conductance in regions perpendicular to the THz field direction and that this enhancement progressively expands toward regions parallel to the field. Pore-formation time decreases with increasing field strength and is shorter when the external field direction aligns with the transmembrane potential, highlighting strong orientation and dose dependence of THz-induced permeabilization.

## Molecular Perturbations to Higher-Order Bioeffects

Although a large number of studies have reported the effects of THz radiation at the cellular level and higher biological levels, as shown in Table 3, there is a clear lack of comparability. Some studies have further attempted to establish cross-scale links between molecular-scale perturbations and cellular phenotypes to explain how localized structural and dynamic changes gradually translate into observable biological effects. Biomolecular structure and dynamics underpin molecular recognition, transport, signaling, and energy conversion. THz-induced changes in conformational populations, hydration, or noncovalent interactions therefore represent intermediate events rather than biological endpoints. To generate higher-order effects, these perturbations must alter functional processes such as ligand binding, ion transport, membrane organization, or gene regulation and then propagate through signaling, metabolism, and intercellular communication. THz bioeffects should thus be understood as context-dependent, multiscale transformations rather than the linear accumulation of microscopic changes.

**Table 3.** Representative biological responses of different cell types to terahertz radiation

Cell type	Study model	Frequency	Dose	Duration	Main bioeffects	Refs.
Stem and progenitor cells	Mouse mesenchymal stem cells	Broadband pulses center at ~10 THz; 1 kHz	Mean ~1 mW/cm^2^	2, 6, or 9 h	Lipid droplet-like structures appeared after 6 h; prolonged exposure altered gene expression and favored adipogenic differentiation.	[123]
	Human embryonic stem cells (hESM01)	2.3 THz; narrowband pulses	Mean ~0.14 W/cm^2^; peak ~4 kW/cm^2^	1 h	Mild up-regulation of ~1% of genes, mainly related to neuroectodermal functions; no detectable DNA damage, chromosomal abnormalities, or changes in proliferation or morphology.	[191]
	Human induced pluripotent stem cells	Broadband THz pulses; 1 kHz	Maximum field ~0.5 MV/cm^1^	1 h	Cell viability and pluripotency were preserved, but cytoskeletal, cell-cycle and differentiation-related gene networks were altered.	[192]
	Human and mouse neural stem cells	0.22 THz	25–50 mW/cm^2^	5–10 min	Proliferation decreased, whereas apoptosis and DNA-damage markers increased; human cells were more sensitive.	[193]
	Reprogrammed human neural progenitor cells	Broadband 0.1–3 THz; strong-field pulses	Peak 21 GW/cm^2^;	30 min	No evident genotoxicity or abnormalities in proliferation or neuronal–glial differentiation.	[194]
Neural and glial cells	Primary mouse hippocampal neurons	0.1 THz; continuous wave	33 mW/cm^2^	20 min;	Differential expression of 165 genes, mainly associated with synaptic function, lipid metabolism, and nucleosome regulation.	[195]
	Primary rat hippocampal neurons	0.12 or 0.157 THz	Mean power 10 or 50 mW	10, 30 min	Cell viability, apoptosis, organelle structure, and amino-acid neurotransmitter release showed frequency-dependent changes.	[196]
	Primary rat hippocampal neurons	28.0–36.1-THz ; pulses	6 W/cm^2^	Scanning exposure for 3 d	Exposure at 34.5 THz promoted neurite elongation and branching and increased PSD95 expression.	[197]
Skin, ocular and vascular cells	Human dermal fibroblasts and HaCaT keratinocytes	0.106 THz; continuous wave	0.04–2 mW/cm^2^	2, 8, 24 h	No DNA strand breaks, micronucleus formation, or abnormal proliferation.	[42]
	Human dermal fibroblasts and HaCaT keratinocytes	0.380 or 2.520 THz	0.03–0.9 mW/cm^2^	2, 8 h	No detectable DNA or chromosomal damage and no observable change in proliferation.	[37]
	Human fetal fibroblasts (HFFF2)	0.1–0.15 THz; pulses	Mean ~0.4 mW/cm^2^	20 min	No evident DNA strand breaks, apoptosis, or telomere changes; micronuclei and anaphase bridges increased slightly.	[39]
	Human dermal fibroblasts	2.52 THz; continuous wave	84.8 mW/cm^2^	5–80 min	Culture temperature increased by ~3 °C, inducing a strong heat-shock response and reduced viability after prolonged exposure.	[198]
	Human corneal epithelial cells (HCE-T)	0.12 THz; continuous wave	5 mW/cm^2^	24 h	No increase in micronuclei or morphological abnormalities and no changes in Hsp27, Hsp70, or Hsp90α expression.	[199]
	Human umbilical vein endothelial cells	2.52 THz; continuous wave	100 mW/cm^2^	10, 30 min	Proliferation was unchanged, whereas migration, tube formation, and VEGF expression and secretion increased.	[144]
Blood and immune cells	Human peripheral blood lymphocytes	0.1 THz; continuous wave	0.031 mW/cm^2^	1, 2, 24 h	Chromosomal aneuploidy and asynchronous centromere replication increased after 2 and 24 h.	[200]
	Human peripheral blood mononuclear cells	0.14 THz; continuous wave	Up to ~10 mW/cm^2^	15 min	Viability decreased in monocytes, B cells and T cells, with distinct sensitivities among cell subsets.	[201]
Tumour cells	Rat C6 glioma cells	0.12–0.18 THz; continuous wave	3.2 mW/cm^2^	1–5 min	Apoptosis increased with exposure duration, indicating time-dependent cytotoxicity.	[163]
	Human SH-SY5Y neuroblastoma cells	0.1 THz; continuous wave	33 mW/cm^2^	30 min	Mitochondrial morphology and dynamics changed; respiratory-chain enzyme activity increased, but overall mitochondrial respiration declined.	[164]
	Human SK-N-BE (2) neuroblastoma cells	Broadband 0.1–3 THz; strong-field pulses	Peak 21 GW/cm^2^	30 min	No detectable DNA damage or abnormalities in proliferation or differentiation.	[194]
	HeLa cervical cancer cells	0.46 THz; 10-ms pulses at 1 Hz	Peak ~600 mW/cm^2^	30, 60 min	F-actin increased, contractile rings persisted and some cells arrested during telophase.	[202]
	4T1 mouse and MCF-7 human breast cancer cells	33 THz; 300 ns pulses at 100 kHz	Total power ~5 mW	Repeated scanning for up to 21 d	Telomerase activity decreased, telomeres shortened, and markers of senescence, apoptosis, and DNA double-strand breaks increased.	[181]
	Human MCF-7 breast cancer cells	Broadband 0.1–24 THz; 1-kHz pulses	496.8–573 mW/cm^2^	1–4 h	Apoptosis increased with exposure duration and intensity, reaching 98.6% after 4 h, accompanied by DNA double-strand breaks.	[180]
	MCF-7, LNCaP, Jurkat, and Ramos cells	0.14 THz; continuous wave	Up to ~10 mW/cm^2^	15 min	Effects on viability and metabolic activity were strongly cell-type dependent.	[201]

THz, terahertz; VEGF, vascular endothelial growth factor; PSD95, postsynaptic density protein 95

Protein conformational dynamics provide several candidate links between molecular perturbation and physiology. At 34.5 THz, reduced Fe^3+^ binding to transferrin was associated with lower cellular iron uptake, diminished Fenton chemistry and lipid peroxidation, and reduced ferroptotic injury in intestinal ischemia–reperfusion [35]. In neural systems, 34-THz exposure weakened glutamate–GluA2 interactions, suppressed anterior cingulate cortex hyperexcitability, and alleviated pain and anxiety-like behavior [97]. By contrast, 34.5-THz stimulation in a posttraumatic stress disorder model enhanced glutamate-NR2B receptor interactions, synaptic plasticity, and cognition [31]. These divergent outcomes indicate that THz effects depend on receptor structure, interfacial modes, exposure conditions, and circuit state, rather than uniformly strengthening or weakening molecular binding.

Changes in protein assembly and aggregation may influence neurodegenerative disease by reshaping proteostasis. In vitro, 34.88-THz radiation altered Aβ β-sheet structure and slowed fibrillization [90]. In animal models, 33-THz binocular stimulation improved cognition and sleep, activated hippocampal cAMP-c-BDNF signaling, restored cholinergic function, and reduced microglial activation [177]. Related studies reported reduced Aβ burden, Tau hyperphosphorylation, and cognitive impairment after THz or mid-infrared exposure [178,179]. However, these molecular and organism-level findings remain largely parallel lines of evidence and do not yet establish that behavioral recovery is caused directly by altered Aβ conformation or aggregation.

Higher-order nucleic acid effects depend not only on DNA duplex structure but also on chromatin accessibility, histone binding, telomere maintenance, and other nucleoprotein processes. THz-induced regulation of nucleic acids is therefore unlikely to arise solely from hydrogen-bond disruption or local strand opening. Zhou et al. [180] showed that high-field THz radiation induced DNA double-strand breaks and activated downstream apoptotic pathways in human epithelial cells, illustrating how structural damage can be translated into cell-fate decisions through canonical stress signaling. By contrast, Alexandrov et al. [122] proposed that 2.52-THz radiation alters promoter breathing dynamics, reshapes differentiation-related transcription in mouse mesenchymal stem cells, and promotes adipogenesis. However, promoter opening was not directly measured, and its necessity for transcriptional reprogramming was not established. Sun et al. [99] reported that 83-THz radiation strengthened DNA-histone interactions, reduced chromatin accessibility at selected glycolytic and migration-related loci, suppressed gene expression, and decreased liver metastasis in a xenograft model. Yin et al. [181] further found that prolonged 33-THz exposure reduced telomerase activity in breast cancer cells and, after repeated proliferation, induced telomere shortening, DNA damage, senescence, and apoptosis, thereby decreasing tumorigenicity in mice. These findings define plausible routes from nucleic acid perturbation to gene regulation and cell fate, but direct causality remains limited by the absence of continuous multilevel measurements, target blockade, and rescue experiments. Nucleic acids and nucleoprotein complexes should therefore be considered candidate response nodes rather than confirmed primary targets.

Ion channels and their membrane environment currently provide one of the clearest links between molecular dynamics and cellular function. THz-induced changes in channel conformation can rapidly alter ion flux, membrane excitability, intracellular signaling, and transcription. Sun et al. reported that 42.5-THz radiation perturbed carboxyl groups and hydration networks within the CaV1.2 SF, enhanced Ca^2+^ permeability, and promoted cAMP response element-binding protein phosphorylation and c-Fos expression [36]. This provides a plausible route by which local pore dynamics are translated into cellular signaling. Similarly, 34.5-THz irradiation activated the PGC-1α-NRF1/2-TFAM pathway through enhanced Ca^2+^ signaling, promoting mitochondrial biogenesis and energy metabolism [182]. In the mouse auditory cortex, 53.6-THz stimulation increased neuronal firing and Ca^2+^ activity and accelerated associative learning by approximately 50% [34].

K^+^ channels can transmit molecular perturbations to neural circuits and behavior by reshaping action potentials and neuronal input–output relationships. Peng et al. [142] showed that 36-THz stimulation increased K^+^ conductance through Kv1.2 channels, accelerated action-potential repolarization, reduced the excitability of anterior cingulate cortex pyramidal neurons, and alleviated neuropathic pain. Similarly, exposure near 53.6 THz enhanced voltage-gated K^+^ currents, accelerated repolarization, altered neuronal gain, and modulated sensorimotor behavior in zebrafish [135].

## Challenges and Future Directions

Despite its low photon energy and inability to directly cleave covalent bonds, THz radiation may regulate biological processes through nonthermal mechanisms, including the selective excitation of low-frequency collective motions, perturbation of molecular dynamics, and reorganization of noncovalent interaction networks. These advances offer important opportunities for molecular modulation strategies and precision therapeutic interventions, while highlighting major challenges in moving the field from phenomenological observations toward mechanistic consensus and translational application.

### Standardizing THz exposure conditions

Conflicting findings in THz bioeffects research largely reflect poor comparability among exposure conditions. THz-induced responses span multiple spatial and temporal scales, and even modest differences in frequency, field strength, pulse structure, exposure duration, or sample geometry can produce distinct physical perturbations. Moreover, molecular responses are often governed by the local stimulus at the target rather than by macroscopic average parameters (Fig. 8A). Standardization is therefore essential for establishing mechanistic consensus and robust dose–response relationships. This requires both minimum reporting standards and a multiscale mapping between platform-level outputs and local molecular exposure, forming the basis of a traceable molecular dosimetry framework [183].

**Fig. 8.**
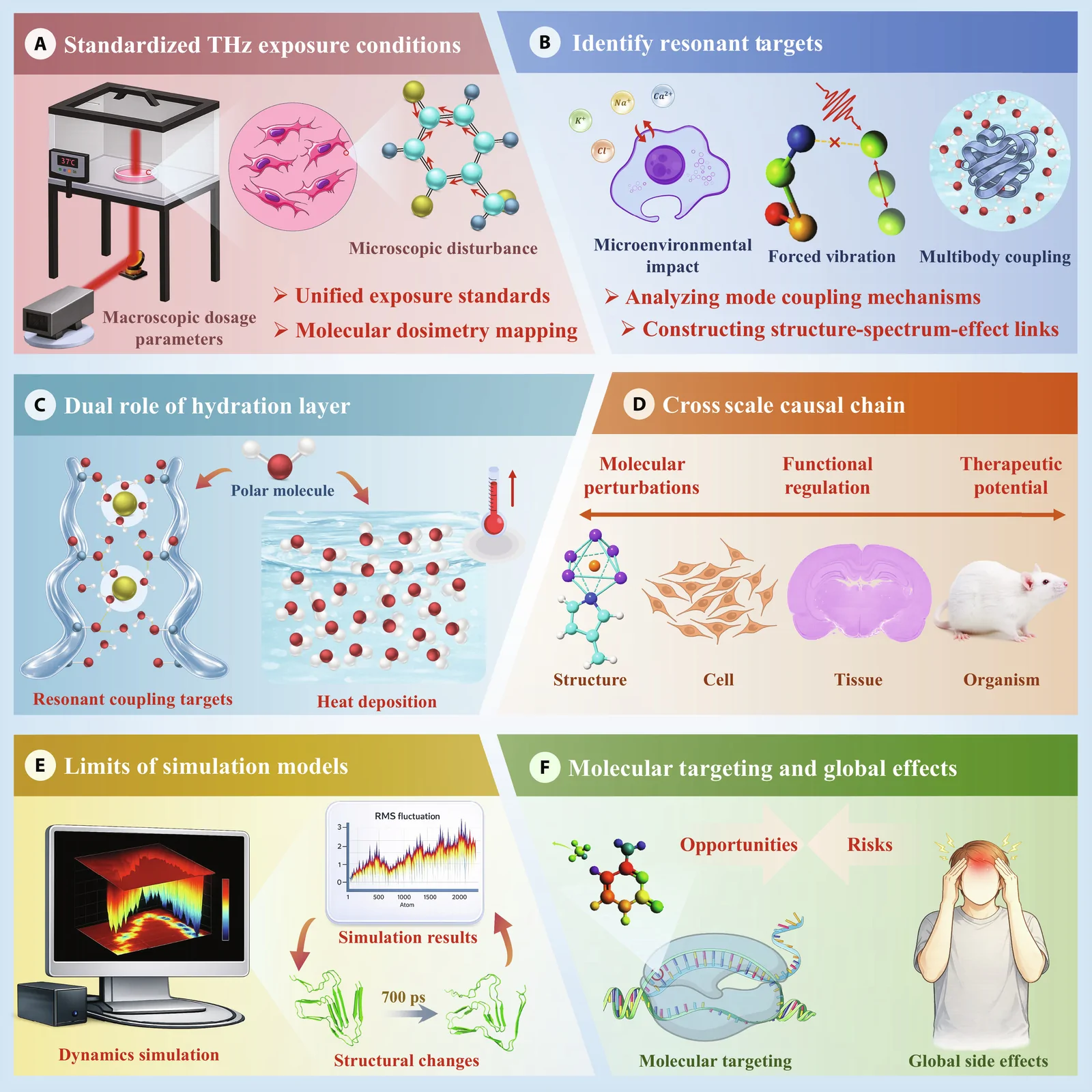
Issues and challenges in terahertz (THz) biological effect research. (A) The lack of standardized dosimetry and exposure protocols limits the comparability and reproducibility of results across studies. (B) The many-body coupling nature of biological systems complicates the precise identification of molecular targets that resonantly couple to THz radiation. (C) The dual role of the hydration layer in THz-induced biological effects remains incompletely understood, particularly in distinguishing nonthermal from thermal contributions. (D) Causal links between molecular-level perturbations and higher-level cellular, tissue, and organ responses remain poorly defined, posing a major challenge for cross-scale interpretation. (E) Current simulation models often rely on simplified representations of biological environments, limiting their ability to accurately capture THz-induced effects in realistic systems. (F) The balance between molecular targeting and system-level effects remains unclear, and the associated risks are not yet well defined.

Future studies should report radiation-source parameters, exposure geometry, sample configuration, and environmental conditions in a standardized manner. Reference samples, harmonized exposure chambers, and interlaboratory validation would further improve reproducibility. Electromagnetic propagation, dielectric measurements, and heat-transfer modeling should then be integrated to map source output onto local fields and energy deposition at tissue, cellular, and molecular scales. Combined with in situ field measurements and high-resolution thermometry, this approach could establish cross-platform molecular dosimetry for dose–response analysis and safety assessment.

### Who is the “resonant target”?

Frequency-selective responses suggest that THz radiation may couple with characteristic molecular modes to reshape molecular energy landscapes. However, as shown in Fig. 8B, attributing such responses to specific molecular resonances remains difficult. Biomolecules operate in crowded environments where conformational fluctuations, hydration dynamics, and intermolecular interactions are strongly coupled [184]. Their modes are also highly sensitive to local conditions, leading to frequency shifts across systems. In addition, nonresonant forcing can generate responses that resemble resonant excitation. The central challenge is therefore to establish a closed evidence chain linking molecular structure, spectral response, dynamics, and function.

This requires moving beyond single-frequency observations toward systematic frequency-response analysis. Continuous or high-density frequency scans should be performed under matched absorption and temperature conditions, with adjacent and nonspecific frequencies as controls. Spectroscopy and multiscale simulations can then identify candidate modes, whereas site-directed mutation, isotope substitution, ligand competition, or conformational restriction can test whether mode shifts are accompanied by predicted functional changes. Such a structure-spectrum-dynamics-function framework would shift the field from empirical frequency screening toward testable and predictive target identification.

### Defining water-mediated effects

The high water content of biological systems inevitably couples molecular perturbations to water-mediated energy deposition, complicating mechanistic attribution (Fig. 8C) [185]. Macroscopic temperature measurements cannot fully resolve transient and spatially heterogeneous energy distributions near molecular targets; the absence of bulk heating therefore does not exclude local thermal deposition or microscale hotspots. Distinguishing hydration-mediated dynamical reorganization from energy absorption across relevant spatial and temporal scales remains a major challenge.

Water should not be treated simply as background interference. Instead, bulk, interfacial, and confined water should be characterized according to their degree of restriction and dynamical properties. Temperature-matched controls, hydration modulation, isotope substitution, and time-resolved spectroscopy can be used to compare physical absorption and biological responses across hydration states. Computationally, local electromagnetic fields, heat transport, hydration dynamics, and biomolecular conformational changes should be integrated into multiscale models that trace energy transfer from bulk water to molecular interfaces. This would transform hydration from an uncontrolled confounder into a quantifiable mechanistic variable.

### Missing cross-scale causal chains

Although THz bioeffects have been reported across multiple scales, most evidence focuses on a single scale (Fig. 8D). How transient molecular perturbations are amplified, filtered, or recoded at the mesoscopic scale through signal transduction and homeostatic feedback, and how subsequently translated into epigenetic, transcriptional, and phenotypic changes, remains poorly understood. A key challenge is to determine when an initial perturbation produces a specific, sustained biological effect rather than a nonspecific stress response.

The solution is not simply to measure more endpoints but to establish continuous, time-resolved observations within a unified exposure system. Studies should follow the sequence of proximal molecular responses, signaling-network changes, cell-state transitions, and tissue-level outcomes. Target mutation, pathway inhibition, phenotypic mimicry, and functional rescue should be used to test the necessity, contribution, and mediating role of key nodes. Integrating time-resolved structural methods, live-cell imaging, and single-cell multiomics could further reveal how initial perturbations decay, amplify, or become reprogrammed within regulatory networks, enabling causal and predictive cross-scale models.

### Boundary of realism in theory and simulation

Computational approaches are indispensable for resolving transient and nonequilibrium molecular responses, but their conclusions are constrained by model assumptions. Simplified systems often omit realistic protonation states, ionic composition, hydration structure, and exposure conditions, whereas force fields, water models, sampling time, and system size limit the description of polarization, collective motion, and slow conformational transitions. In particular, the simulation results cannot be correlated with functional phenotypes without resolving intermediate events or scale transformations (Fig. 8E).

Future modeling should match model resolution to the scientific question, combining atomistic, coarse-grained, and mesoscopic approaches where appropriate. Realistic protonation states, ionic environments, hydration conditions, and external fields should be incorporated, while controlled approximations, such as effective potentials and continuum descriptions, can reduce computational cost without removing key physics. Analyses should also move beyond generic structural descriptors toward mechanistically informative variables, including conformational-state populations, site accessibility, binding affinity, enzymatic activity, and electrophysiological output. Replicate simulations, sensitivity analyses, and cross-model validation are essential for defining model applicability and the evidential limits of computational inference.

### “Molecular targeting” may produce “global side effects”

THz radiation has been reported to influence DNA methylation, DNA breathing, membrane permeability, and ligand dissociation or stabilization under nominally nonthermal conditions. Yet, in hydrated, many-body biological systems, such molecular responses are embedded within extensive regulatory networks and may be amplified through signaling and feedback into broader biological outcomes. Conversely, homeostatic compensation, repair, and adaptation may buffer early perturbations. Biological risk therefore cannot be inferred from macroscopic temperature rise alone [186].

For example, short-term epigenetic modulation may appear controllable, but its long-term consequences for transcriptional programs and cell states remain uncertain. Likewise, ligand destabilization or stabilization without spatial confinement may produce off-target and systemic effects (Fig. 8F). Future studies should define mechanism-specific response windows and safety margins, extending safety assessment beyond bulk heating to include local molecular dose, effect duration, reversibility, and long-term biological outcomes. Multiscale phenotyping, spatiotemporal monitoring, and longitudinal follow-up will be required to balance intended effects against potential risks and to support the preclinical development of THz biomedical technologies.

## Conclusion and Prospects

Research on THz bioeffects is revealing a noninvasive paradigm for molecular regulation, in which the frequency characteristics of THz radiation may enable selective resonant coupling to modulate biomolecular conformation and function under primarily nonthermal, nonionizing conditions, suggesting potential applications in controlled molecular regulation and biointervention. Over the past 2 decades, studies have demonstrated that THz exposure can elicit specific, dose-dependent responses across multiple levels of biological organization and produce bidirectional modulation in some case. Notably, advances in enhancing motor and cognitive function, promoting tissue development, and regulating cellular activity highlight its potential for interventions in complex diseases, including cancer, neurodegenerative disorders, and inflammatory conditions, as well as relevance to research on complex conditions such as cancer, neurodegenerative disorders, and inflammatory diseases. As understanding of macroscopic phenotypic complexity deepens, research is shifting from phenomenological observation to mechanistic analysis, aiming to elucidate the nonequilibrium and coordinated responses induced by THz perturbation across spatial and temporal scales. Moreover, extending investigations to higher frequency ranges reduces background interference from water relaxation, enabling studies to more directly probe the intrinsic resonant responses of biomolecules and their regulatory mechanisms.

Despite important progress in understanding molecular-level mechanisms, a systematic understanding of how THz perturbations act on local biomolecular targets and subsequently propagate to higher-order biological effects remains lacking, and computational predictions have yet to be fully reconciled with experimental observations into a coherent chain of evidence. In addition, heterogeneity in exposure conditions, model systems, and observational scales complicates the development of unified conceptual frameworks and mechanistic models. Experimental studies are further constrained by strong water absorption and limited penetration depth, difficulties in quantifying local dose, insufficient spatiotemporal resolution of current instrumentation, and potential unintended perturbations introduced by the experimental interventions themselves, all of which complicate mechanistic attribution and validation. Future studies should therefore strengthen the bidirectional integration of computational prediction and experimental validation within standardized exposure conditions and traceable dosimetry frameworks. This will require interdisciplinary efforts spanning biophysics, cell biology and biochemistry, together with complementary experimental approaches, omics analyses and targeted perturbations, to cross-validate key molecular events and determine how these perturbations propagate across cellular and tissue scales, ultimately establishing a closed and testable multiscale mechanistic framework. Building on this foundation, standardized exposure protocols, clearer criteria for distinguishing nonthermal effects, well-defined dose windows and safety thresholds, combined with real-time monitoring and dynamic feedback control, will be essential for advancing THz technology from laboratory exploration toward controllable, predictable, and clinically translatable biomedical applications, providing new conceptual and methodological foundations for precision therapeutic interventions in complex diseases.
